# Targeting PML in triple negative breast cancer elicits growth suppression and senescence

**DOI:** 10.1038/s41418-019-0407-5

**Published:** 2019-10-01

**Authors:** Leire Arreal, Marco Piva, Sonia Fernández, Ajinkya Revandkar, Ariane Schaub- Clerigué, Josep Villanueva, Amaia Zabala-Letona, Mikel Pujana, Ianire Astobiza, Ana Rosa Cortazar, Ivana Hermanova, Laura Bozal-Basterra, Amaia Arruabarrena-Aristorena, Jana R. Crespo, Lorea Valcarcel-Jimenez, Patricia Zúñiga-García, Francesc Canals, Veronica Torrano, Rosa Barrio, James D. Sutherland, Andrea Alimonti, Natalia Martin-Martin, Arkaitz Carracedo

**Affiliations:** 1 CIC bioGUNE, Derio, Spain; 2 CIBERONC, Derio, Spain; 3 Institute of Oncology Research (IOR) and Oncology Institute of Southern Switzerland (IOSI), Bellinzona, CH 6500 Switzerland; 4 Faculty of Biology and Medicine, University of Lausanne (UNIL), Lausanne, CH 1011 Switzerland; 5 Vall d´Hebron Institute of Oncology (VHIO), Barcelona, Spain; 6 Biochemistry and Molecular Biology Department, University of the Basque Country (UPV/EHU), Bilbao, Spain; 7 IKERBASQUE, Basque Foundation for Science, Bilbao, Spain

**Keywords:** Oncogenes, Tumour-suppressor proteins

## Abstract

Oncogene addiction postulates that the survival and growth of certain tumor cells is dependent upon the activity of one oncogene, despite their multiple genetic and epigenetic abnormalities. This phenomenon provides a foundation for molecular targeted therapy and a rationale for oncogene-based stratification. We have previously reported that the Promyelocytic Leukemia protein (PML) is upregulated in triple negative breast cancer (TNBC) and it regulates cancer-initiating cell function, thus suggesting that this protein can be therapeutically targeted in combination with PML-based stratification. However, the effects of PML perturbation on the bulk of tumor cells remained poorly understood. Here we demonstrate that TNBC cells are addicted to the expression of this nuclear protein. PML inhibition led to a remarkable growth arrest combined with features of senescence in vitro and in vivo. Mechanistically, the growth arrest and senescence were associated to a decrease in MYC and PIM1 kinase levels, with the subsequent accumulation of CDKN1B (p27), a trigger of senescence. In line with this notion, we found that PML is associated to the promoter regions of MYC and PIM1, consistent with their direct correlation in breast cancer specimens. Altogether, our results provide a feasible explanation for the functional similarities of MYC, PIM1, and PML in TNBC and encourage further study of PML targeting strategies for the treatment of this breast cancer subtype.

## Introduction

Breast cancer exemplifies the potential of gene expression profiling to classify the disease into molecular subtypes [1–3]. However, these classifications do not inform about the molecular mediators of tumor progression and metastasis in each subtype of breast cancer. To address this question, we and others have defined genes and pathways that are relevant to breast cancer progression, metastasis, and resistance to therapy [4–6]. The Promyelocytic Leukemia protein (PML), the essential component of the PML nuclear bodies (PML-NBs), induces apoptosis and inhibits angiogenesis and cell cycle progression in cancer, thus complying with the definition of a tumor suppressor [7, 8]. Paradoxically, PML exerts a prosurvival role conferring a selective advantage in chronic myeloid leukemia and specific solid tumors [6, 9–15]. In breast cancer, PML regulates aggressiveness and metastatic features through the control of the stem cell gene, *SOX9*, and the Hypoxia-inducible factor 1 alpha (HIF1α) signaling [13, 15]. Moreover, the regulation of cancer-initiating cell (CIC) and metastatic potential is restricted to PML high-expressing estrogen receptor-negative breast tumors, predominantly triple negative breast cancer (TNBC).

The concept that the perturbation of a driver cancer gene can exert an exacerbated tumor suppressive response in tumor cells is defined as “oncogene addiction” and provides a rationale for molecular targeted therapy [16]. Senescence is a stress response that involves a stable cell growth arrest as well as an adaptive process to reduce energy consumption for cell division or differentiation and therefore assure the survival and viability of the cell [17, 18]. Senescence is induced in vitro by different stimuli including DNA damage, oxidative stress, oncogene activation, mitochondrial dysfunction, or chemotherapy [17, 18]. Cyclin-dependent kinase inhibitor family (CDKi) is a key regulator of the senescence response, predominantly through p53-p21 and/or p16-RB axes [17, 18]. To a lesser extent, CDKN1B (p27) has been reported to participate in the activation of the senescence response, in conditions where p21 and/or p16 are not active [19, 20]. Of note, PML is required for a fully functional senescence response upon oncogene activation in tumors where it functions as a tumor suppressor. In addition, the PML-NBs coordinate the activation of p53 and the formation of the senescence-associated heterochromatin foci (SAHF) [21–23].

TNBC exhibits increased levels and activity of various oncogenes, including MYC and PIM1 [24–27]. Importantly, these genes regulate metabolic and signaling activities in this breast tumor subtype, and they represent an attractive therapeutic vulnerability [24, 26–28]. Whereas similarities exist among the reported activities of MYC, PIM1, and PML, their functional association remains obscure. In this study, we demonstrate that TNBC cells that express high PML levels are addicted to the nuclear protein, and its targeting elicits a growth suppressive response that encompasses MYC and PIM1 downregulation and the activation of p27-dependent senescence.

## Results

### PML silencing induces senescence and prevents tumor growth in vivo

**Fig. 1:**
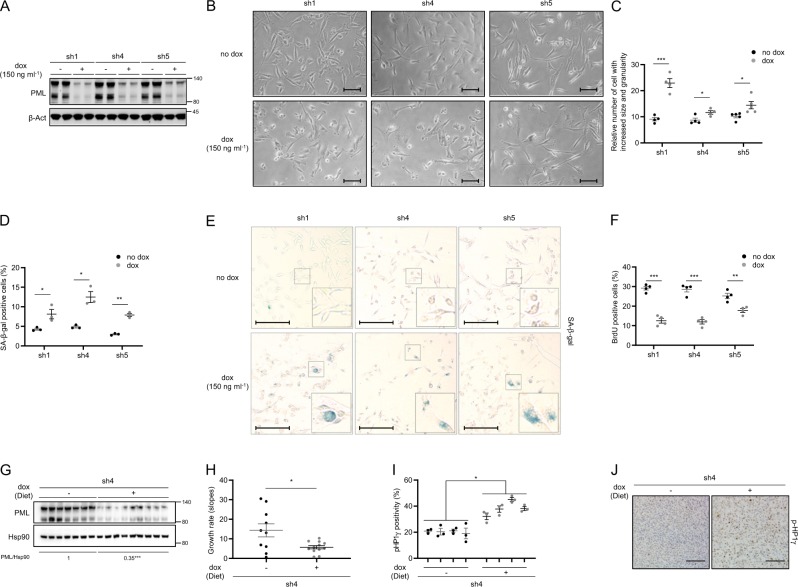
PML silencing induces senescence. Effect of doxycycline-inducible (150 ng ml^−1^; 3 + 3 days) PML silencing (sh1, sh4, and sh5) on PML protein expression (**a**, representative of at least three experiments), on the morphology (**b**, representative images, scale bar, 50 μm), on cell size and granularity (**c**, FACS analysis, sh1 and sh4, *n* = 4, sh5, *n* = 5), on the number of senescent cells (**d**; *n* = 3, representative images of SA-β-Galactosidase assay, scale bar 50 μm (**e**)) and on the number of BrdU positive cells (**f**, *n* = 4) in MDA-MB-231 cells. Impact of doxycycline-inducible PML silencing (sh4) of established MDA-MB-231 xenografts on PML protein expression (**g**), on tumor growth rate represented as the growth rate of each tumor (**h**, sh4 no dox, *n* = 10; sh4 dox, *n* = 12; growth rate was inferred from the linear regression calculated for the progressive change in tumor volume of each individual tumor during the period depicted in Supplementary Fig. 1p) and on number of senescent cells measured by p-HP1γ staining (**i**, sh4 no dox, *n* = 4; sh4 dox, *n* = 4); representative images of p-HP1γ positive cells, scale bar 100 μm (**j**) of the tumors. Error bars represent s.e.m. p, *p*-value (*p < 0.05, **p < 0.01, ***p < 0.001). One-tailed Student's *t*-test was used for cell line data analysis (**c**, **d**, **f**) and one-tailed Mann–Whitney *U*-test for xenografts (**h**, **i**). sh1, sh4, and sh5: shRNA against *PML*, dox: doxycycline, SA-β-gal: senescence-associated beta-galactosidase, BrdU: bromodeoxyuridine, p-HP1γ: phospho-heterochromatin protein-1 gamma, molecular weight markers (kDa) are shown to the right

The identification of PML as a novel target in aggressive breast cancer tumors [13, 15] prompted us to investigate the molecular consequences of its inhibition in an established cell culture. To this end, we generated and validated three PML-targeting doxycycline-inducible and two constitutive short hairpin RNAs (shRNAs) (Fig. 1a and Supplementary Fig. 1a) [15]. PML silencing triggered a robust morphological change in PML-high expressing cells, MDA-MB-231 (Fig. 1b and Supplementary Fig. 1b), characterized by a significant increase in size (FSC-A) and granularity (SSC-A) analyzed by FACS (Fig. 1c and Supplementary Fig. 1c). These changes in morphology were indicative of a senescence response. Indeed, the evaluation of senescence-associated β-galactosidase (SA-β-gal) activity in both inducible and constitutive systems confirmed this notion (Fig. 1d, e and Supplementary Fig. 1d, e) in MDA-MB-231 cells. Senescence is defined as an irreversible cell cycle arrest. Indeed, we could confirm the cell cycle arrest upon PML genetic inhibition, by means of BrdU analysis (Fig. 1f) and crystal violet cell number assay (Supplementary Fig. 1f, g) and that it was not due to an increase in apoptosis (Supplementary Fig. 1h). Of note, arsenic trioxide (ATO) did not elicit a senescence phenotype (Supplementary Fig. 1i). This compound exerts a biphasic effect on PML; first favors the formation of the PML NBs and then the degradation of PML. Therefore, the inability of ATO to recapitulate PML silencing could be due to its molecular mode of action.

We monitored additional features that were reported for certain types of oncogene-induced senescence [29]. On the one hand, proteomics analysis of the supernatant of these cells indicated that PML silencing resulted in a distinct secretome, without signs of a canonical SASP (senescence-associated secretory phenotype) (Supplementary Fig. 1j–l and Supplementary Table 1). On the other hand, we ascertained the formation of SAHF. We could not confirm the existence of SAHF neither at the level of chromatin condensation nor the formation of macroH2A1.1 foci (Supplementary Fig. 1m). Lamin B1 loss is a senescence-associated biomarker [30, 31]. We demonstrated that in our system PML loss induced a decrease in Lamin B1 protein levels (Supplementary Fig. 1n–o).

Of note, PML regulates oxidative stress responses [7, 32]. We ruled out that reactive oxygen species (ROS) elevation drives senescence in our system since PML loss does not induce its accumulation (Supplementary Fig. 1p).

Breast CIC capacity is reduced upon PML knockdown in TNBC cells (with high PML expression), as we demonstrated in limiting dilution assays with MDA-MB-231 cells [15]. Here, we hypothesized that the activation of senescence would result in a tumor suppressive response in established tumors, where the contribution of CIC is negligible. To test this notion, MDA-MB-231 cells were injected in the flank of immunocompromised mice, and once the tumors were established (reaching a volume of 25–130 mm^3^) doxycycline was administered in the food pellets to induce PML silencing. In agreement with the response observed in vitro, xenograft growth was curbed upon PML knockdown (Fig. 1g, h and Supplementary Fig. 1q–r) and senescence increase was confirmed by means of p-HP1γ staining (Fig. 1i, j) [33]. Our data suggest that PML silencing in a PML-high expressing TNBC cell line triggers a senescence response with a partial presence of classical markers of this process.

### p27 is the driver in PML loss-induced senescence

**Fig. 2:**
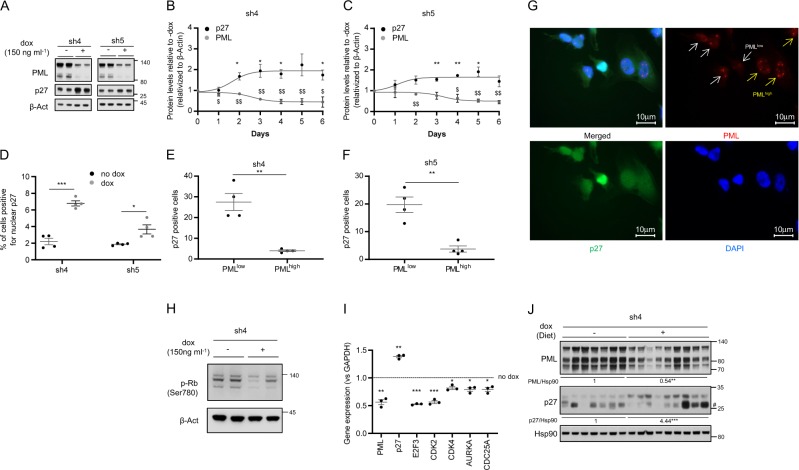
p27 is induced after PML silencing. **a** Effect of doxycycline-inducible (150 ng ml^−1^; 3 + 3 days) PML silencing (sh4, sh5) on p27 and PML protein expression (representative of at least three experiments) on MDA-MB-231 cells. **b**, **c** Quantification of p27 and PML protein levels along 6 days of doxycycline-inducible PML silencing on MDA-MB-231 cells (*n* = 3) with two different shRNAs. **d** Immunofluorescence quantification of nuclear p27 positive cells upon PML inducible silencing on MDA-MB-231 cells (*n* = 4). **e**–**g** Immunofluorescence quantification of the correlation of p27 positive cells and PML levels in these cells (**e**–**f**) and representative images of p27 and PML staining (**g**) upon doxycycline-inducible PML silencing in MDA-MB-231 cells (*n* = 4). **h** Effect of doxycycline-inducible (150 ng ml^−1^; 3 + 3 days) PML silencing (sh4) on RB phosphorylation (Ser780) (representative of three experiments) on MDA-MB-231 cells. **i** Expression of p27-related cell cycle genes upon PML inducible silencing in MDA-MB-231 cells (*n* = 3). **j** Impact of doxycycline-inducible PML silencing (sh4) on p27 and PML protein expression on established MDA-MB-231 xenografts. Error bars represent s.e.m. p, *p*-value (*^/$^*p* < 0.05, **^/$$^*p* < 0.01, ***^/$$$^*p* < 0.001). One-tailed one-sample *t*-test (**b**, **c**, **i**) and one-tailed Student’s *t*-test were used for cell line data analysis (**d**–**f**). sh4 and sh5: shRNA against *PML*, dox: doxycycline. ^#^Unspecific band. Molecular weight markers (kDa) are shown to the right

Senescence is executed and sustained at the molecular level through the activation of growth suppressors, including p53 and the cyclin-dependent kinase (CDKs) inhibitors p21, p16, and p27 [17, 20, 29]. Since MDA-MB-231 cells harbor loss of p16 and p53 mutation [34, 35], we proposed p27 as a candidate to drive PML silencing-induced senescence in our cell system. Importantly, p27 protein levels were increased upon both inducible (Fig. 2a and Supplementary Fig. 2a) and constitutive (Supplementary Fig. 2b) PML silencing with all the shRNA tested in MDA-MB-231 cells. Moreover, we observed that the induction of p27 protein levels occurred as soon as 2 days following PML inactivation and it was maintained up to 6 days of PML silencing (Fig. 2b, c and Supplementary Fig. 2c–f).

The function of p27 is controlled by changes in its levels along with its compartmentalization within the cell [36]. To confirm the functionality of accumulated p27 in PML-silenced cells, we quantified p27 nuclear localization by immunoflourescence. As predicted, PML silencing elicited an increase of nuclear p27 in cells with the three inducible shRNA tested (Fig. 2d and Supplementary Fig. 2g). Since the effect of shRNAs was analyzed in a pooled culture, it is plausible that there would be heterogeneity in PML levels across cells within the culture dish. We therefore evaluated whether the increase in nuclear p27 was ascribed to cells with a profound decrease in PML immunoreactivity. We established an immunofluorescence score based on previous studies [6, 15] (PML low = 0–4 dots; PML high = more than 4 dots per cell nuclei). In line with our previous results, we observed a significant inverse association between PML immunoreactivity and p27 nuclear staining (Fig. 2e–g and Supplementary Fig. 2h). Moreover, the elevated activity of p27 upon PML silencing was consistent with the increase in its mRNA levels, the blockade of Retinoblastoma protein (Rb) phosphorylation and the reduced transcription of downstream regulated cell cycle-related genes (Fig. 2h, i and Supplementary Fig. 2i). In agreement with the results observed in vitro, p27 accumulation was recapitulated upon PML knockdown in vivo (Fig. 2j).

**Fig. 3:**
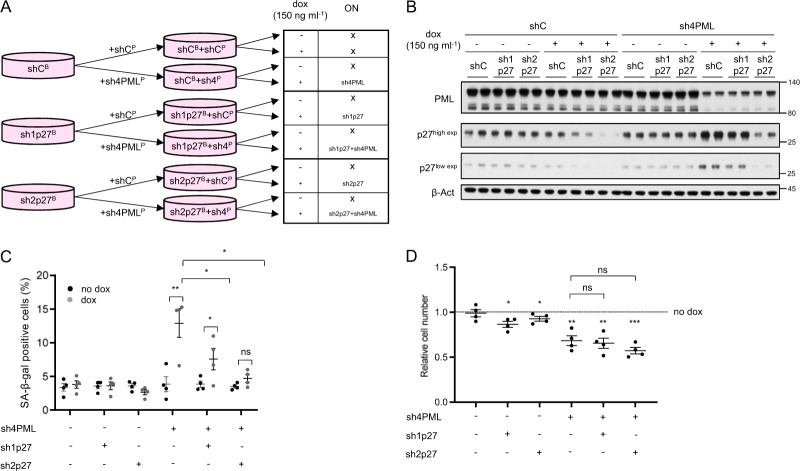
p27 is the driver in PML loss-induced senescence. **a** Experimental design for inducible p27 silencing (sh1p27 and sh2p27, B: blasticidin selection) alone or in combination with inducible PML silencing (sh4, P: puromycin selection) in MDA-MB-231 cells. **b** p27 and PML protein levels upon doxycycline inducible silencing of either p27 or PML or both in MDA-MB-231 cells (representative of three experiments). **c** Effect on the number of senescent cells (*n* = 4) upon p27 and/or PML inducible silencing in MDA-MB-231 cells. **d** Effect on the relative cell number (*n* = 4) upon p27 and/or PML inducible silencing in MDA-MB-231 cells. Error bars represent s.e.m. p, *p*-value (*p < 0.05, **p < 0.01, ***p < 0.001). One-tailed Student's *t*-test (**c**) and one-tailed one-sample *t*-test (**d**) were used for cell line data analysis. shC: scramble shRNA, Dox: doxycycline, SA-β-gal: senescence-associated beta-galactosidase. Molecular weight markers (kDa) are shown to the right

Our results reveal that PML silencing in TNBC cells with high expression of the nuclear protein triggers a senescence response associated to p27 accumulation. To ascertain the causal contribution of p27 to the execution of the senescence response, we silenced p27 in MDA-MB-231 cells concomitantly with PML silencing, using inducible (Fig. 3a, b) or constitutive (Supplementary Fig. 3a) shRNA systems. Preventing p27 accumulation upon PML loss hampered the induction of senescence in a dose-dependent manner according to the potency of the shRNA against p27 (Fig. 3c and Supplementary Fig. 3b). Our results demonstrate that PML loss elicits a senescence response mediated by the upregulation of p27 in PML high expressing TNBC cells.

Although senescence is a major driver of growth arrest, we noticed that the amount of SA-β-Gal positivity upon PML silencing was not comparable to the extent of growth arrest detected (Fig. 1 and Supplementary Fig. 1). Taking advantage of our capacity to ablate senescence by silencing p27, we ascertained the contribution of this response to the cell number reduction. Importantly, the growth arrest caused by PML silencing was not recovered by blunting senescence (Fig. 3d and Supplementary Fig. 3c), thus suggesting that an additional mechanism may be involved.

### MYC and PIM1 are regulated by PML in TNBC

**Fig. 4:**
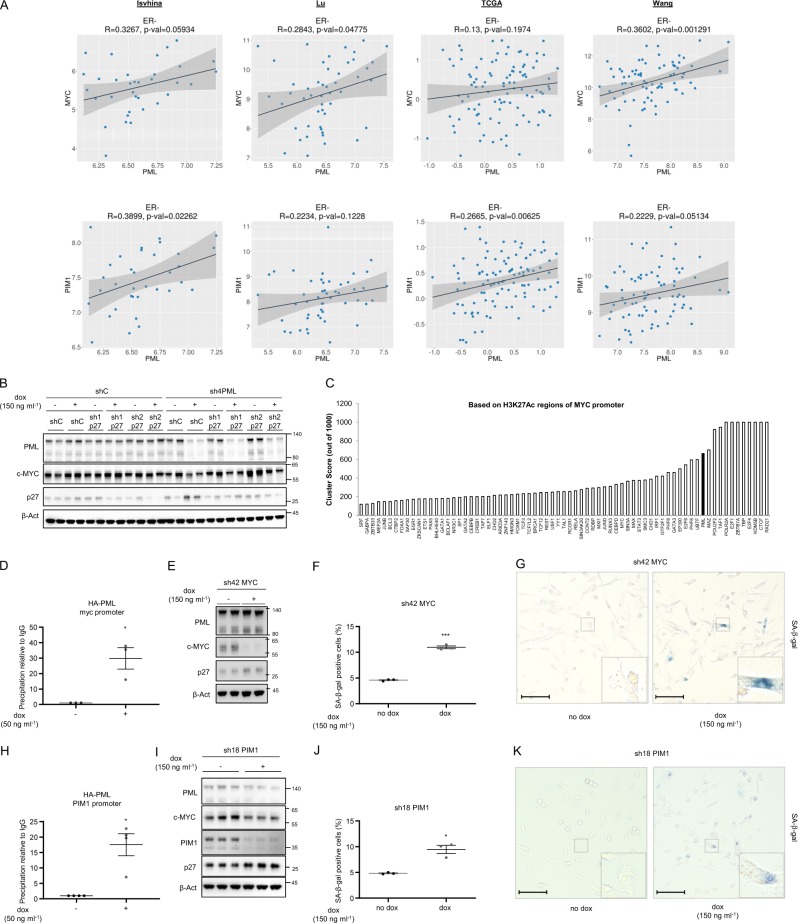
PML regulates MYC and PIM1 expression in TNBC. **a** Correlation analysis between PML and MYC (top panels) and between PML and PIM1 (bottom panels) mRNA levels in ER negative tumor specimens of the indicated breast cancer datasets. Sample sizes: Ivshina (*n* = 34), Lu (*n* = 49), TCGA (*n* = 117) and Wang (*n* = 77). **b** p27, MYC, and PML protein levels upon doxycycline inducible silencing of either p27 or PML or both in MDA-MB-231 cells (representative of three experiments). **c** Cluster score of DNA-binding proteins in MYC promoter region using ENCODE database. **d** MYC promoter region abundance in chromatin immunoprecipitation (ChIP) of exogenous HA-PMLIV using HA-tag antibody in MDA-MB-231 cells after induction with 50 ng ml^−1^ doxycycline for 3 days (*n* = 3). Data were normalized to IgG (negative-binding control). **e** p27, MYC, and PML protein levels upon doxycycline inducible silencing of MYC (sh42) in MDA-MB-231 cells (representative of three experiments). Effect on the number of senescent cells (*n* = 3) (**f**) and representative images, scale bar 50 μm, (**g**) upon MYC inducible silencing in MDA-MB-231 cells. **h** PIM1 promoter region abundance in chromatin immunoprecipitation (ChIP) of exogenous HA-PMLIV using HA-tag antibody in MDA-MB-231 cells after induction with 50 ng ml^−1^ doxycycline for 3 days (*n* = 4). Data were normalized to IgG (negative-binding control). **i** p27, MYC, PIM1, and PML protein levels upon doxycycline inducible silencing of PIM1 (sh18) in MDA-MB-231 cells (representative of three experiments). **j**–**k** Effect on the number of senescent cells (*n* = 3) and representative images, scale bar 50 μm, (**k**) upon PIM1 inducible silencing in MDA-MB-231 cells. Error bars represent s.e.m. p, *p*-value (*p < 0.05, ***p < 0.001). One-tailed one sample *t*-test (**d**, **h**) and one-tailed student's *t*-test (**f**, **j**) were used for cell line data analysis. shC: Scramble shRNA, Dox: doxycycline, SA-β-gal: senescence-associated beta-galactosidase. Molecular weight markers (kDa) are shown to the right

Our data demonstrate that preventing p27 accumulation is not sufficient to rescue the growth arrest caused by PML loss. We reasoned that the mechanism through which PML was regulating growth arrest, p27 accumulation and senescence might depend on a larger growth-regulatory program. Interestingly, the oncogenic axis comprised with MYC and PIM1 kinase shares many similarities with PML concerning its activity in TNBC. MYC and PIM1 are upregulated in TNBC [24, 26, 27] and inhibit p27 accumulation and function [37]. In addition, MYC regulates metabolic functions attributed to PML, including fatty acid β oxidation [6, 10, 38, 39] and its inhibition induces cellular senescence in lymphoma, osteosarcoma, and hepatocellular carcinoma [40]. With this data in mind, we first evaluated the association of PML, MYC, and PIM1 in breast cancer. We found a significant direct correlation in various breast cancer transcriptomics datasets. This association was evident in two out of four datasets for MYC-PML and four out of four for PIM1-PML, when accounting all breast cancer subtypes (Supplementary Fig. 4a). Since the effect of these genes is restricted to tumors that lack hormone receptors, we refined the analysis by focusing on estrogen receptor (ER) negative tumors. In this scenario, the correlation was recapitulated in various datasets (Fig. 4a).

We next aimed at deconstructing the molecular regulation of PML, MYC, and PIM1. First, we monitored the impact of PML silencing on MYC abundance. As predicted, inducible PML shRNA activation resulted in a remarkable decrease in MYC protein and mRNA levels in vitro and in vivo (Supplementary Fig. 4b–h) in two PML high expressing cells, MDA-MB-231 and MDA-MB-468. Of note, in line with our senescence results (Supplementary Fig. 1i), ATO did not alter the abundance of p27 and MYC (Supplementary Fig. 4i–j).

We next asked to which extent MYC downregulation was retained in cells devoid of p27-dependent senescence response. To address this question, we checked MYC expression upon PML/p27 double silencing in MDA-MB-231 cells. The decrease in MYC expression upon PML loss was not recovered with p27 silencing (Fig. 4b), thus providing a feasible explanation for the lack of rescue in growth capacity.

We have previously shown that PML can regulate gene expression in line with its association with discreet promoter regions [15]. Since PML silencing resulted in reduced MYC mRNA levels (Supplementary Fig. 4d, h), we interrogated MYC promoter in silico using ENCODE [41]. We found PML among the proteins with highest confidence DNA-binding score in MYC promoter region (Fig. 4c, cluster score: 527). We performed chromatin immunoprecipitation (ChIP) analysis of ectopically expressed PML and confirmed that PML is in the vicinity of MYC promoter (Fig. 4d). To ascertain if MYC silencing recapitulated the effect of PML inhibition, we used a validated shRNA targeting this oncogene (sh42) [42, 43] and confirmed that MYC silencing resulted in increased p27 levels (Fig. 4e and Supplementary Fig. 4k), senescence (Fig. 4f, g) and growth arrest (Supplementary Fig. 4l).

In the last few years, an important body of work has demonstrated that PIM1 is an important partner of MYC function in prostate cancer and TNBC [24, 26]. Moreover, PIM1 can regulate MYC transcriptional signature and p27 [24, 26]. We monitored the impact of PML on PIM1 expression and function. PML loss resulted in a decrease in PIM1 gene expression in two PML high expressing cells, MDA-MB-231, and MDA-MB-468 (Supplementary Fig. 4m–n). Importantly, we confirmed that PML is in close proximity to PIM1 promoter by ChIP analysis (Fig. 4h, cluster score: 383 in ENCODE). We hypothesized that loss of PIM1 would further impact on MYC function and recapitulate the aforementioned PML and MYC phenotype. We silenced PIM1 using a validated shRNA (sh18) [24] and corroborated that the targeting of PIM1 led to decrease in MYC abundance, increase in p27 levels, senescence, and growth arrest in MDA-MB-231 cells (Fig. 4i–k and Supplementary Fig. 4o–q). Altogether, our results provide strong support for the role of MYC-PIM1 axis supporting PML-elicited TNBC growth and preventing the accumulation of p27 and senescence.

### PML loss-elicited growth suppression in breast cancer is selective of high PML expressing TNBC

**Fig. 5:**
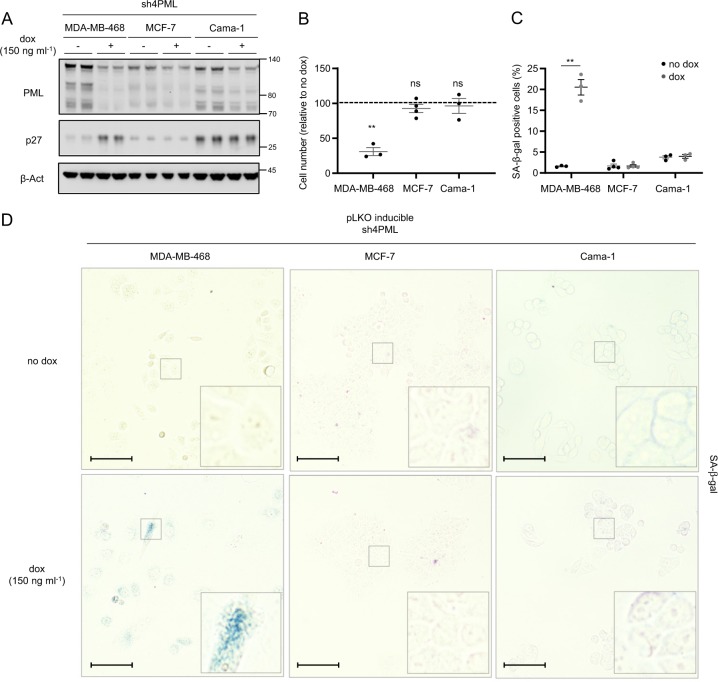
The antiproliferative program elicited by PML loss is restricted to PML-high expressing TNBC cells. Effect of doxycycline-inducible (150 ng ml^−1^; 3 + 3 days) PML silencing (sh4) on PML and p27 protein expression (**a**, representative of three experiments), on cell number (**b**, MDA-MB-468 and Cama-1, *n* = 3, MCF7, *n* = 4) and on the number of senescent cells (**c**, MDA-MB-468 and Cama-1, *n* = 3, MCF7, *n* = 4; representative images of SA-β-galactosidase positive cells (**d**)) in MDA-MB-468, MCF-7, and Cama-1 cells, scale bar 50 μm. Error bars represent s.e.m. p, *p*-value (*p < 0.05, **p < 0.01, ***p < 0.001, ns: not significant). One-tailed one sample *t*-test (**b**) and one-tailed Student's *t*-test were used for cell line data analysis (**c**). Dox: doxycycline, SA-β-gal: senescence-associated beta-galactosidase. Molecular weight markers (kDa) are shown to the right

The inactivation of a single oncogene can compromise the development and survival of tumor cells despite their genetic or epigenetic abnormalities [16]. We have previously reported that high PML levels in TNBC are required for adequate CIC function [15]. Here, the data presented support the notion that the bulk of tumor cells in a TNBC with elevated PML is “addicted” to the expression of the protein. In turn, acute depletion of the nuclear protein results in growth arrest and senescence. To ascertain whether the “addiction” was restricted to TNBC cells, we took advantage of various breast cancer cell lines belonging to distinct subtypes with differing levels of PML. A second TNBC cell line (MDA-MB-468) that presented high levels of PML protein was compared with ER+ cells [15] (Fig. 5a). Silencing of PML in MDA-MB-468 cells elicited a remarkable growth arrest, which was not detected in the ER+ cells Cama-1 and MCF7 (Fig. 5b and Supplementary Fig. 5a–c). In line with this notion, Cama-1 and MCF7 cells did not exhibit neither the reduction in MYC expression nor the induction of p27-dependent senescence, as compared with MDA-MB-468 cells (Fig. 5c, d and Supplementary Fig. 5d–g). Moreover, the morphological changes induced by the loss of PML were only present in TNBC cells (Supplementary Fig. 5h). Since PML silencing resulted in a distinct secretory phenotype (albeit not a canonical SASP), we monitored the secretome of the ER + cell line Cama-1. In agreement with our prior data, unsupervised clustering based on the secretome was ineffective in segregating experimental conditions according to PML status. Similarly, principal component analysis and hierarchical clustering reinforced the notion that Cama-1 are refractory to PML level perturbation (Supplementary Fig. 5i–k and Supplementary Table 2).

## Discussion

PML has been a paradigmatic tumor suppressor since its discovery [7, 8, 44]. A variety of molecular activities directly support its reported capacity to prevent many of the hallmarks of cancer, including the induction of apoptosis and the inhibition of proliferation or angiogenesis [7]. Molecular partners such as p53 have reinforced this notion. However, the discovery of tumoral contexts, where the presence of PML is required has broadened the picture of the roles of this nuclear protein in disease. Depletion of PML impairs the self-renewal activity in the leukemic stem cell from chronic myeloid leukemia [9, 10]. This phenotype is the consequence of both cell autonomous (the hyper-activation of mTOR complex1 and the reduction in PPARδ-fatty acid oxidation activity [9, 10]) and non-cell autonomous activities (regulation of the mesenchymal stem cell in the leukemic niche [45]), which trigger symmetric commitment and the loss of the CIC compartment.

The regulation of CIC activity was recently translated to solid tumors. To date, glioma and a subset of breast cancers exhibit PML-dependent self-renewal activity [14, 15], whereas other tumors, such as ovarian cancers or some experimental models of hepatocarcinoma development, exhibit a broad tumor suppressive response upon PML inhibition [11, 12]. PML expression is selectively exacerbated in a subset of breast tumors (TNBC) [6, 13, 15]. Yet, we lack basic understanding around the impact of PML on the function of this cell subtype. In this study, we demonstrate that PML depletion in the bulk of TNBC cells in culture and in vivo triggers a tumor suppressive response consisting on growth arrest and the activation of senescence.

PML has been previously related to the regulation of the senescence response [21]. However, the majority of studies associate PML expression to the execution of this growth suppressive response upon the activation of oncogenes or replicative stress [11]. Experimentally, ectopic PML expression triggers senescence, and, conversely, PML deletion bypasses the senescence response elicited by the oncogenic form of RAS, thus enabling transformation [46–48]. Mechanistically, PML supports p53 activity and participates in the formation of SAHF [21]. In turn, PML loss bypasses the senescence response. Paradoxically, our results indicate that, in cancer cells with high dependence on PML expression, its inhibition also triggers a senescence response that lacks canonical SASP and SAHF. This phenomenon might have been overlooked in prior studies due to the lack of data at the time on the role of PML favoring cancer cell function in specific tumor subsets. Of note, the activation of senescence in non-transformed fibroblasts upon PML depletion adds complexity to already extensive portfolio of PML activities [49].

Oncogene addiction [16] is perceived as an attractive opportunity in the era of targeted therapies. Our results are consistent with PML addiction in TNBC cells, even if this protein cannot be formally considered an oncogene. The data produced by us and others [6, 13, 15] argue in favor of a molecular make up in this subtype of breast cancer that requires the presence of PML in high doses, as opposed to estrogen receptor-positive tumor cells. In this regard, the control of MYC and PIM1 expression by PML provides a feasible explanation for the accumulation of p27 and the induction of senescence when PML is silenced. To which extent the relationship between PML, MYC, and PIM1 is operative in other tumor types becomes now an exciting question to address. Since PML lacks a dedicated domain to recognize and bind discreet DNA sequences, the existence of yet unidentified PML-interacting transcription factors that enable this regulatory mode warrants further research.

The results obtained in this study represent a conceptual leap in how we perceive the role of PML in TNBC, and suggest that targeting this nuclear protein can be beneficial at multiple levels, including impairing the CIC function [15], blunting hypoxia signaling [13], and triggering a senescence response. The quantification of the relative relevance of each PML effector pathway in the overall activity of PML could open new opportunities to apply the biology of PML-regulated TNBC function for breast cancer treatment.

## Materials and methods

### Cell culture

MDA-MB-231, MDA-MB-468, MCF-7, and Cama-1 cell lines were obtained from the American Type Culture Collection (ATCC, Manassas, VA, USA) or from Leibniz-Institut—Deutsche Sammlung von Mikroorganismen und Zellkulturen GmbH (DMSZ) who provided an authentication certificate. None of the cell lines used in this study was found in the database of commonly misidentified cell lines maintained by ICLAC and NCBI Biosample. Cell lines were routinely monitored for mycoplasma contamination and quarantined while treated if positive. MDA-MB-231 and MCF-7 cells were maintained in DMEM media, MDA-MB-468 were maintained in RPMI media, and Cama-1 were maintained in DMEM-F12 media, all supplemented with 10% (v/v) foetal bovine serum and 1% (v/v) penicillin-streptomycin.

### Generation of stable cell lines

293FT cells were used for lentiviral production. Lentiviral vectors expressing shRNAs against human *PML* and *p27* from the Mission® shRNA Library were purchased from Sigma‐Aldrich. Cells were transfected with lentiviral vectors following standard procedures, and viral supernatant was used to infect cells. Selection was done using puromycin (2 μg ml^−1^; P8833, Sigma) for 48 h or blasticidin (10 μg ml^−1^; Cat. 15205, Sigma) for 5 days. As a control, a lentivirus with scrambled shRNA (shC) was used. Short hairpins sequence: shC: CCGGCAACAAGATGAAGAGCACCAACTCGAGTTGGTGCTCTTCATCTTGTTGTTTTT; sh1PML (TRCN0000003865): CCGGCAATACAACGACAGCCCAGAACTCGAGTTCTGGGCTGTCGTTGTATTGTTTTT; sh4PML (TRCN 0000003867): CCGGGCCAGTGTACGCCTTCTCCATCTCGAGATGGAGAAGGCGTACACTGGCTTTTT; sh5PML (TRCN 0000003868): CCGGGTGTACCGGCAGATTGTGGATCTCGAGATCCACAATCTGCCGGTACACTTTTT; sh1p27 (TRCN 0000039928): CCGGGTAGGATAAGTGAAATGGATACTCGAGTATCCATTTCACTTATCCTACTTTTTG; sh2p27 (TRCN 0000039930): CCGGGCGCAAGTGGAATTTCGATTTCTCGAGAAATCGAAATTCCACTTGCGCTTTTTG. sh42MYC (TRCN0000039642): CCGGCCTGAGACAGATCAGCAACAACTCGAGTTGTTGCTGATCTGTCTCAGGTTTTTG. sh18PIM1 (TRCN0000010118): CCGGACATCCTTATCGACCTCAATCCTCGAGGATTGAGGTCGATAAGGATGTTTTTT.

Sub-cloning of shC, sh1PML, sh4PML, sh5PML, and sh42myc into pLKO-Tet-On-Puromycin vector was done introducing *AgeI* and *EcoRI* in the 5′end of top and bottom shRNA oligos respectively (following the strategy provided by Dr. Dmitri Wiederschain [50], Addgene plasmid: 21915). Sub-cloning of shC, sh1p27, sh2p27, and sh18PIM1 into pLKO-Tet-On-Blasticidin was done following the same procedure. Puromycin resistance cassette was replaced by Blasticidin cassette following Gibson assembly strategy.

### Reagents

Doxycycline (Cat. D9891, Sigma) was used at 150 ng ml^−1^ to induce the expression of shRNA from pLKO-Tet-On vectors. Doxycycline-mediated inducible shRNA expression was performed by treating cell cultures for 72 h with the antibiotic (150 ng ml^−1^) and then seeding for cellular or molecular assays in the presence of doxycycline for three more days (unless otherwise specified). ATO (Cat. A1010, Sigma-Aldrich) was prepared at a concentration of 100 mM in NaOH 1 N and subsequently diluted to 0.1 mM in PBS for a working solution. ATO was used at 150 nM for 6 days as indicated in figure legends.

### Cell growth analysis and size measurement by FACS

Cell number quantification was done with crystal violet as reported [5]. For FACS analysis MDA-MB-231 cells were trypsinized and resuspended in PBS to be analysed based on their size (FSC) and granularity (SSC) using a BD FACSCanto^TM^ II (BD Biosciences) flow cytometer upon PML doxycycline-inducible silencing. Data represented in Fig. 1c correspond to the sum of Q1 + Q2 + Q3 populations selected as in Supplementary Fig. 1c. Data were analysed using the FlowJo software; cell populations were selected for each shRNA (no dox condition) and differences quantified for increasing size and granularity.

### Senescence associated-β-galactosidase detection

To quantify the number of senescent cells, constitutive or inducible PML/MYC/PIM1/p27 silencing cells was performed as described previously and cells were seeded in 24-well plates in duplicate. An overnight incubation with the senescence detection kit (QIA117, Calbiochem) was performed and SA-β-Gal activity was revealed and quantified (three areas per well, more than 200 cells per condition). The number of senescent cells in each area was relativized to the number of total cells counted per area. Cells were seeded in plates or glass cover slips to acquire images with EVOS® cell imaging station (×20 magnification objective).

### Western blotting, immunofluorescence and BrdU

Western blot analysis was carried out as previously described [5]. Briefly, cells were seeded on six-well plates. Cell lysates were prepared with RIPA buffer (50 mM TrisHCl pH 7.5, 150 mM NaCl, 1 mM EDTA, 0.1% SDS, 1% Nonidet P40, 1% sodium deoxycholate, 1 mM Sodium Fluoride, 1 mM sodium orthovanadate, 1 mM beta-glycerophosphate and protease inhibitor cocktail; Roche). The following antibodies were used for Western blotting: rabbit polyclonal anti-PML, 1:1000 dilution (Cat: A301–167A, Bethyl laboratories), mouse monoclonal anti-p27[Kip1], 1:1000 dilution (Cat: 610242, BD Biosciences), mouse monoclonal anti-beta-ACTIN, 1:2000 dilution (Cat: 3700, Cell Signaling), rabbit polyclonal Hsp90, 1:2000 dilution (Cat: 4874, Cell Signaling), rabbit polyclonal c-Myc, 1:1000 dilution (Cat: 13987, Cell Signaling), rabbit polyclonal PIM1, 1:1000 dilution (ab75776, Abcam), rabbit polyclonal Lamin B1 (ab133741, Abcam), rabbit monoclonal anti-cleaved PARP (Asp214), 1:1000 dilution (Cat: 5625, Cell Signaling), rabbit polyclonal anti-cleaved caspase 3 (Asp175), 1:1000 dilution (Cat: 9661, Cell Signaling), mouse monoclonal anti-α-Tubulin (66031–1-Ig, Proteintech), 1:2500 dilution, rabbit monoclonal anti-phospho-Rb (Ser780) 1:1000 dilution (Cat: 9307, Cell Signaling). After standard SDS-PAGE and Western blotting techniques, proteins were visualized using the ECL on iBright™ CL1000 Imaging System (Cat: A32749, Invitrogen). Densitometry-based quantification was performed using ImageJ software. Uncropped scans are provided in Supplementary Fig. 6.

For immunofluorescence, cells were seeded on glass cover slips in 24-well plates, cells were fixed with 4% para-formaldehyde (15 min), PBS (three times wash), 1% Triton X-100 (5 min), PBS (three3 times wash), 10% goat serum (1 h) and anti-PML antibody 1:100 dilution (catalog A301–167A; Bethyl laboratories), anti-p27[Kip1] antibody 1:100 dilution (Cat: 610242, BD Biosciences) and anti-macroH2A1.1 antibody 1:100 (Cat: 12455, Cell Signaling) were added ON (4 °C) in goat serum. Cover slips were washed with PBS three times and incubated with secondary antibodies (anti-rabbit Alexa488, anti-rabbit Alexa594, anti-mouse Alexa488, and anti-mouse Alexa594; Invitrogen-Molecular Probes) for 1 h (room temperature). Cover slips were washed with PBS three times, and DAPI added to stain nuclei (10 min), followed by mounting with ProLong™ Gold Antifade Mountant (Cat: P36930, Invitrogen). Immunofluorescence images were obtained with AxioImager D1 microscope (Zeiss) or with a confocal microscopy (Leica SP8) with ×63 objectives. At least three different areas per cover slip were quantified.

For BrdU analysis cells were seeded as for immunofluoresce. Prior to fixing, cells were incubated in the presence of BrdU (3 ug ml^−1^). Cells were fixed with 4% para-formaldehyde (15 min), PBS (three times wash) and DNA exposed with 2 M HCl (5 min), PBS (3 times wash) and 0,1 M sodium borate. After that, PBS (three times wash), 1% Triton X-100 (5 min), PBS (three times wash), 10% goat serum (1 h), and monoclonal anti-BrdU (MoBU-1) antibody 1:100 dilution (Cat: B35128, Invitrogen) was added ON (4 °C) in goat serum. Cover slips were washed three times with PBS and incubated with secondary antibodies (anti-mouse Alexa594; Invitrogen-Molecular Probes) for 1 h (room temperature). Cover slips were washed three times with PBS and DAPI added to stain nuclei (10 min), followed by mounting with ProLong™ Gold Antifade Mountant (Cat: P36930, Invitrogen). Images were obtained with an AxioImager D1 microscope (Zeiss). At least three different areas per cover slip were quantified.

### Quantitative real-time PCR

Cells were seeded as for western blot. Total RNA was extracted from cells using NucleoSpin RNA isolation kit from Macherey-Nagel (ref: 740955.250). Complementary DNA was produced from 1 µg of RNA using Maxima™ H Minus cDNA Synthesis Master Mix (Cat# M1682, Invitrogen). Taqman probes were obtained from Applied Biosystems. Amplifications were run in a Viia7 or QS6 Real-Time PCR Systems (Applied Biosystems) using the following probes: PML (Hs00971694_m1, cat: 4331182). For p27 (CDKN1B), MYC, PIM1, CDK2, CDK4, E2F3, AURKA, and CDC25A amplification, Universal Probe Library (Roche) primers and probes were employed (p27, For: ccctagagggcaagtacgagt, Rev: agtagaactcgggcaagctg, probe: 39; MYC, For: gctgcttagacgctggattt, Rev: taacgttgaggggcatcg, probe: 66; PIM1, For: atcaggggccaggttttc, Rev: gggccaagcaccatctaat, probe: 13; CDK2, For: aaagccagaaacaagttgacg, Rev: gtactgggcacaccctcagt, probe 77; CDK4, For: gtgcagtcggtggtacctg, Rev: aggcagagattcgcttgtgt, probe 25; E2F3, For: ggtttcggaaatgcccttac, Rev: gatgaccgctttctcctagc, probe 40; AURKA, For: gcagattttgggtggtcagt, Rev: tccgaccttcaatcatttca, probe 79; CDC25A, For: cgtcatgagaactacaaaccttga, Rev: tctggtctcttcaacactgacc, probe 67). All quantitative PCR with reverse transcription data presented were normalized using GAPDH (Hs02758991_g1, cat: 4331182) from Applied Biosystems as housekeeping.

### Mice

Xenograft experiments were carried out following the ethical guidelines established by the Biosafety and Welfare Committee at CIC bioGUNE. The procedures employed were carried out following the recommendations from AAALAC. Xenograft experiments were performed as previously described [5], injecting 3·10^6^ cells per tumor, two injections per mouse, one per flank. All mice (female Hsd:Athymic Nude-Foxn1 nu/nu) were inoculated at 8–12 weeks of age. Nineteen days post injection, once tumors were stablished (25–130 mm^3^), mice were fed with chow or doxycycline diet (Research diets, D12100402) until the experimental endpoint.

### p-HP1γ immunohistochemistry

After sacrifice, formalin-fixed paraffin embedded xenograft tissues were stained for p-HP1γ. Tissues were deparaffinized using the standard procedure and unmasking/antigen retrieval was performed using pH 6.0 solution for 20 min at 98 °C in water bath. Tissue sections were stained for p-HP1γ using primary antibody Phospho-HP1γ (Ser83) (Cat. No: 2600, Cell Signaling technologies, 1:200) and secondary antibody Biotinylated antibody Anti-Rabbit (BP-9100, Vector Laboratories, 1:200). This was followed by Vectastain ABC solution incubation (PK-6100, Vector laboratories, 1:150) and DAB staining (SK-4105, Vector laboratories) as per the manufacturer’s protocol. Stained slides were scanned using Leica Aperio AT2 slide scanner. The criteria for senescent staining used for quantification was a very prominent nuclear staining in which the nucleus was bigger in size and its staining was darker brown than the other cells.

### ChIP

ChIP was performed using the SimpleChIP® Enzymatic Chromatin IP Kit (Cat #9003, Cell Signaling Technology, Inc) as reported [15]. DNA quantification was carried out using a Viia7 Real-Time PCR System (Applied Biosystems) with SybrGreen reagents and primers that amplify the predicted PML binding region to MYC promoter (chr8:128748295–128748695) as follows: left primer: CCGGCTAGGGTGGAAGAG, right primer: GCTGCTATGGGCAAAGTTTC and PIM1 promoter (chr6:37137097–37137612) as follows: left primer: ACTCCCTCCGTGACTCATGT, right primer: ACGAGGGTGGTCTTTCTGTG.

### Secretome analysis

Secretomes were prepared as previously described [51]. MDA-MB-231 sh4 PML tet on and Cama-1 sh4 PML tet on cells were pre-induced with doxycycline (150 ng ml^−1^) for 3 days. Three 150 cm^2^ plates where seeded per condition: 4 × 10^6^ cells per plate of non-induced cells and 5 × 10^6^ cells per plate of doxycycline induced cells. After two days, cell supernatants were removed and cells were washed five times: the first two washes were performed with PBS and the last three were made with serum-depleted DMEM. Cells were left to grow for 24 h in serum-depleted DMEM. Doxycicline was maintained (150 ng ml^−1^). Two biological replicates, each with three technical replicates were processed.

After 24 h supernatant was collected and one dish per condition was trypsinized and counted to check cell number and PML expression. The supernatant was first spun at 1000 rpm for 5 min followed by filtration through 0.2 μm filtering bottles. After this, it was concentrated using 10 kDa Amicons; first, 15 mL Amicons (Ref. UCF901024, Merck) were used, followed by 0.5 mL Amicons (Ref. UCF501069, Merck) to get final volumes close to 80 μL. The concentrated secretome was frozen at −20 °C until proteomics analysis. Protein concentration was determined with a Pierce BCA protein assay kit (Thermo Scientific). All samples were digested with trypsin in-solution prior to analysis by liquid chromatography−mass spectrometry (LC−MS). Tryptic digests were analysed by shotgun proteomics using an LTQ Velos-Orbitrap mass spectrometer (Thermo Fisher Scientific, Bremen, Germany). The RAW files of each MS run were processed using Proteome Discoverer (Thermo Fisher Scientific), and MS/MS spectra were searched against the human database of Swiss-Prot using the MASCOT (Matrix Science, London, U.K) algorithm. The results files generated from MASCOT (.DAT files) were then loaded into Scaffold (Proteome Software, Portland, OR), resulting in a nonredundant list of identified proteins per sample achieving a protein false discovery rate (FDR) under 1.0%, as estimated by a search against a decoy database.

### Secretome statistical analysis

Relative spectral counting-based protein quantification analysis was performed on the different samples analyzed using Scaffold. Files containing all spectral counts for each sample and its replicates were generated and then exported to R software for normalization and statistical analysis [52]. All statistical computations were done using the open-source statistical package R. The data were assembled in a matrix of spectral counts, where the different conditions are represented by the columns and the identified proteins are represented by the rows. An unsupervised exploratory data analysis by means of principal components analysis and hierarchical clustering of the samples on the SpC matrix was first performed. Then, the GLM model based on the Poisson distribution was used as a significance test [52]. Finally the Benjamini and Hochberg multitest correction was used to adjust the *p-*values with control on the FDR.

Full information regarding the proteins detected in the secretome analysis can be found in Supplementary Tables 1 and 2.

### ROS analysis

MDA-MB-231 cells with inducible shRNA against PML (sh4) were pre-induced with doxycycline (150 ng ml^−1^) for 3 days. Then, cells were seeded in a six-well plate in triplicate (1.5 × 10^5^ cells/well) maintaining the doxycycline concentration. Two additional wells with non-induced cells were used for positive and negative ROS controls respectively.

After 72 h, 10 µM of 2′, 7′-Dichlorofluorescin diacetate (DCF-DA) (Sigma-Aldrich Ref: 35845) reactive was added to each well and cells were incubated for 30 min. In the last 5 min of the incubation time, 1 M hydrogen peroxide (H_2_O_2_) was added to the positive control well.

Subsequently, cells were washed with PBS and raised from plates employing 500 µL of trypLE reactive (Gibco™ ref: 12563–011). After that, cells were washed twice with abundant PBS to eliminate the excess of DCF-DA reactive and pellets were re-suspended in 500 µL PBS for FACS analysis. Samples were analyzed in FACS CANTO II for green fluorescence.

### Datasets

#### Database normalization

All the datasets used for the data mining analysis were downloaded from GEO and TCGA, and subjected to background correction, log_2_ transformation, and quartile normalization. In the case of using a pre-processed dataset, this normalization was reviewed and corrected if required.

#### Correlation analysis

Pearson correlation test was applied to analyse the relationship between paired genes. From this analysis, Pearson coefficient (R) indicates the existing linear correlation (dependence) between two variables *X* and *Y*, giving a value between +1 and −1 (both included), where 1 is total positive correlation, 0 is no correlation, and −1 is total negative correlation. The *p*-value indicates the significance of this R coefficient.

### Statistical analysis

No statistical method was used to predetermine sample size. The experiments were not randomized. The investigators were not blinded to allocation during experiments and outcome assessment. Data analysed by parametric tests are represented by the mean ± s.e.m. of pooled experiments unless otherwise stated. *n* values represent the number of independent experiments performed or the number of individual mice. For each in vitro independent experiment, technical replicates were used and a minimum number of three experiments were performed to ensure adequate statistical power. In the in vitro experiments, normal distribution was assumed and one sample *t*-test was applied for one component comparisons with control and Student’s t-test for two component comparisons. For in vivo experiments, a non-parametric Mann–Whitney *U*-test was used. Two-tailed statistical analysis was applied for experimental design without predicted result, and one-tail for validation or hypothesis-driven experiments. The confidence level used for all the statistical analyses was of 0.95 (alpha value = 0.05).

## Supplementary information

Suppl Figures and legends

Suppl Table 1

Suppl Table 2
