# Supplementary material for: Targeting PML in triple negative breast cancer elicits growth suppression and senescence
Source: Cell Death Differ. 2019 Oct 1;27(4):1186–99. doi: 10.1038/s41418-019-0407-5 (PMC7104349; doi:10.1038/s41418-019-0407-5)

Supplementary Figure legends

Supplementary Figure 1. (a-b) Effect of PML constitutive silencing (sh4, sh5) on PML protein expression (**a**, representative of at least 3 experiments) and on the morphology (**b**, representative images, scale bar 50μm). (**c**) Schematic representation of one representative experiment of the defined populations represented in main Fig.1c upon inducible PML silencing on MDA-MB-231 cells. **(d-e)** Effect on the number of senescent cells upon constitutive PML silencing (**d**; n=13) and representative images of SA-β-Galactosidase assay in MDA-MB-231 cells, scale bar 50μm (**e**). **(f-g)** Impact on cell number upon either constitutive (**f**, n=12) or inducible (**g**, sh1 and sh5, n=4, sh4, n=7) PML silencing in MDA-MB-231 cells. **(h)** Effect of PML inducible silencing (sh4) on apoptosis using staurosporine (Stp) as a positive control. **(i)** Effect on the number of senescent cells after 150 nM arsenic trioxide treatment during 6 days in MDA-MB-231 cells. **(j-l)** Proteomics analysis of the secretome of MDA-MB-231 cells upon PML inducible silencing: **(j)** PML and tubulin levels in both cells extracts and secretome samples (dash lines indicate samples used in the analysis), **(k)** unsupervised exploratory data analysis by means of principal component analysis and **(l)** heat maps representing the proteins that were significantly over- and under-secreted upon PML silencing in MDA-MB-231 cells. Data analysis was based on spectral count data after exporting it from Scaffold software into R. The GLM model based on the Poisson distribution was used to test significance. Only the proteins with spectral counts of 2, Log2FC of 0.8 and adjusted p-value of 0.05 are present in the heatmap. Columns represent samples; rows are proteins. Red represents proteins that are over-secreted and green represents proteins that are under-secreted. The data rows are centred and scaled to 1 standard deviation prior to produce the heat map. **(m)** Immunofluorescence of macroH2A1.1 and DAPI upon inducible silencing of PML in MDA-MB-231 cells. **(n-o)** Levels of Lamin B1 protein upon PML inducible silencing in MDA-MB-231 cells (**n**, representative of 5 experiments) and **(o)** protein quantification (n=5). **(p)** Effect on ROS production (n=4) after inducible PML silencing in MDA-MB-231 cells. **(q-r)** Impact of inducible PML silencing

on tumour growth **(q)** and tumour weight **(r)** of established MDA-MB-231 xenografts (sh4 no dox, n=10; sh4 dox, n=12). Error bars represent s.e.m. p, p-value (*p< 0.05, **p< 0.01, ***p< 0.001) for figure f and g (* sh4 vs shC or dox; $ sh5 vs shC; # sh1 vs shC). One-tailed Student´s t-test (d, i) and one-tailed one sample t-test (f-g, o-p) were used for cell line data analysis, and one-tailed Mann-Whitney U-test for xenografts (q-r). shC: Scramble shRNA, sh1, sh4 and sh5: shRNA against *PML*, Dox: doxycycline, SA-β-gal: Senescence-associated beta-galactosidase, VC: vehicle control, ATO: arsenic trioxide, ROS: reactive oxygen species. Molecular weight markers (kDa) are shown to the right.

Supplementary Figure 2. (a-b) Protein levels of p27 and PML after either inducible (sh1) (**a**) or constitutive (**b**) PML silencing in MDA-MB-231 cells (representative of 3 experiments). **(c)** Quantification of p27 and PML protein levels along 6 days of doxycycline-inducible PML silencing on MDA-MB-231 cells (n=3) with sh1. **(d-f)** Representative western blots of inducible PML silencing with the 3 different short hairpins during 6 days of induction graphed on main Fig. 2b-c and Suppl. Fig. 2c. **(g-h)** Immunofluorescence quantification of nuclear p27 positive cells (**g**) and correlation of p27 positive cells and PML levels **(h)** (upon PML inducible silencing on MDA-MB-231 cells with sh1). **(i)** Schematic representation of the p27-Rb protein interaction and regulation. Error bars represent s.e.m. p, p-value (*p< 0.05, **p< 0.01, ***p< 0.001). One-tailed Student´s t-test (g-h) and one-sample t-test (c) were used for cell line data analysis. shC: Scramble shRNA, sh1, sh4 and sh5: shRNA against *PML*, Dox: doxycycline, D: day, RB: retinoblastoma protein. CDK: cyclin dependent kinases. Molecular weight markers (kDa) are shown to the right.

Supplementary Figure 3. (a) p27 and PML protein levels after constitutive silencing of either p27 or PML or both in MDA-MB-231 cells (representative of 4 experiments). **(b-c)** Effect on the number of senescent cells (n=4) **(b)** and cell growth (n=3) **(c)** after constitutive p27 and/or PML silencing. Error bars represent s.e.m. p, p-value (*p< 0.05, **p< 0.01, ***p< 0.001 compared with shC or as indicated, ns: not significant). One-tailed Student´s t-test (b) and one-tailed one-sample t-test (c)

were used for cell line data analysis. shC: Scramble shRNA, SA-β-gal: Senescence-associated beta-galactosidase. Molecular weight markers (kDa) are shown to the right.

Supplementary Figure 4. (a) Correlation analysis between PML and MYC (top panels) and between PML and PIM1 (bottom panels) mRNA levels in all breast cancer subtypes of tumor specimens of the indicated breast cancer datasets. Sample sizes: Ivshina (n=249), Lu (n=131), TCGA (n=522) and Wang (n=286). **(b-d)** MYC, p27 and PML protein levels (representative of 3 experiments) **(b)**, quantification of the protein (n=3) **(c)** and MYC gene levels **(d)** after inducible silencing of PML (sh4) in MDA-MB-231 cells (n=3). **(e)** PML, p27 and MYC mRNA levels upon doxycycline-inducible PML silencing (sh4) of established MDA-MB-231 xenografts. (**f-h)** MYC, p27 and PML protein levels (representative of 3 experiments) **(f)**, quantification of the protein (n=3) **(g)** and MYC gene levels **(h)** after inducible silencing of PML (sh4) in MDA-MB-468 cells (n=3). **(i-j)** MYC, p27 and PML protein levels (representative of 5 experiments) **(i)** and quantification of the protein (n=5) **(j)** after 150 nM arsenic trioxide treatment during 6 days in MDA-MB-231 cells. **(k-l)** Protein quantification from Fig. 4e **(k)** and impact in cell number (n=3) **(l)** of inducible MYC silencing (sh42) in MDA-MB-231 cells. **(m-n)** PIM1 gene levels after PML inducible silencing in MDA-MB-231 (n=3) **(m)** and MDA-MB-468 (n=3) **(n)** cells. **(o-q)** Protein quantification from Fig. 4i **(o)**, gene expression levels of PIM1, MYC and p27 **(p)** and impact in cell number (n=3) **(q)** of inducible PIM1 silencing (sh18) in MDA-MB-231 cells. Error bars represent s.e.m. p, p-value (*p< 0.05, **p< 0.01, ***p< 0.001, ns: not significant). One-tailed one sample t-test (c-d, g-h, j-q) was used for cell line data analysis and one-tailed Mann-Whitney U-test for xenografts (e). sh4: shRNA against *PML*, sh42: shRNA against *MYC*, sh18: shRNA against *PIM1*. Dox: doxycycline. VC: vehicle control. ATO: arsenic trioxide. Molecular weight markers (kDa) are shown to the right.

Supplementary Figure 5. (a-c) Impact of PML inducible silencing (sh4) on cell number in MDA-MB-468 **(a)**, MCF-7 **(b)** and Cama-1 **(c)** cell lines. **(d-e)** p27, MYC and PML protein levels **(d)** and

protein quantification **(e)** of PML inducible silencing in MCF-7 cells. **(f-g)** p27, MYC and PML protein levels **(f)** and protein quantification **(g)** of PML inducible silencing in Cama-1 cells. **(h)** Impact of PML inducible silencing (sh4) on cell morphology in MDA-MB-468, MCF-7 and Cama-1 cell lines, scale bar 50μm. **(i-k)** Proteomics analysis of the secretome of Cama-1 cells upon PML inducible silencing: **(i)** PML and tubulin levels in both cells extracts and secretome samples (dash lines indicate samples used in the analysis), **(j)** unsupervised exploratory data analysis by means of principal component analysis and **(k)** heat maps representing the proteins that were significantly over- and under-secreted, upon PML silencing in Cama-1 cells. Data analysis was based on spectral count data after exporting it from Scaffold software into R. The GLM model based on the Poisson distribution was used to test significance. Only the proteins with spectral counts of 2, Log_2FC of 0.8 and adjusted p-value of 0.05 are present in the heat map. Columns represent samples; rows are proteins. Red represents proteins that are over-secreted and green represents proteins that are under-secreted. The data rows are centered and scaled to 1 standard deviation prior to produce the heatmap. Error bars represent s.e.m. p, p-value (*p< 0.05, **p< 0.01, ***p< 0.001). One-tailed one sample t-test was used for cell line data analysis (a-c, e, g). sh4: shRNA against *PML*. Dox: doxycycline. Molecular weight markers (kDa) are shown to the right.

Supplementary Figure 6. Uncropped scans.

Supplemental Table 1. List of differentially secreted proteins upon silencing the PML protein in MDA-MB-231 cells.

Supplemental Table 2. List of differentially secreted proteins upon silencing the PML protein in Cama-1 cells.

Supp. Figure 1

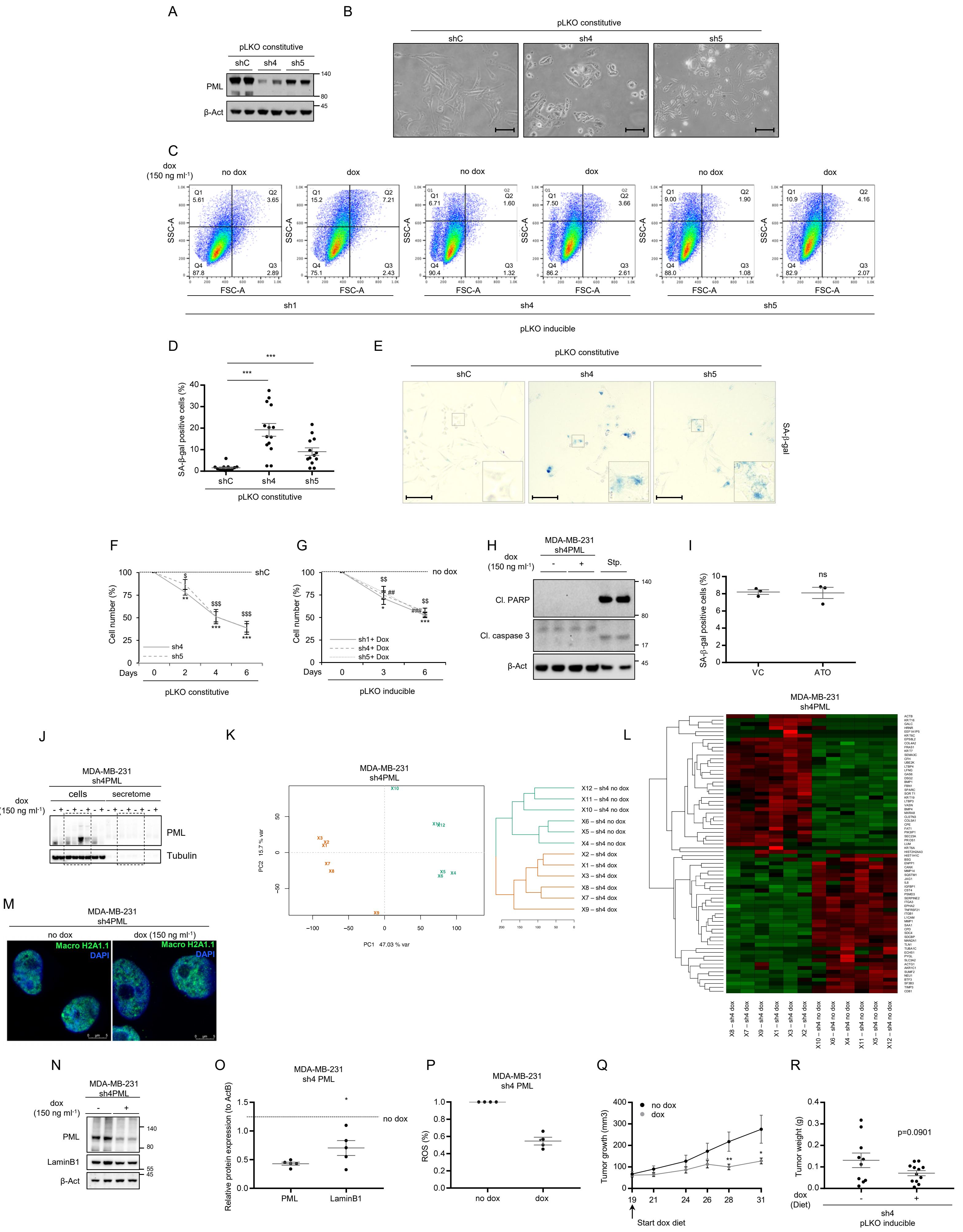

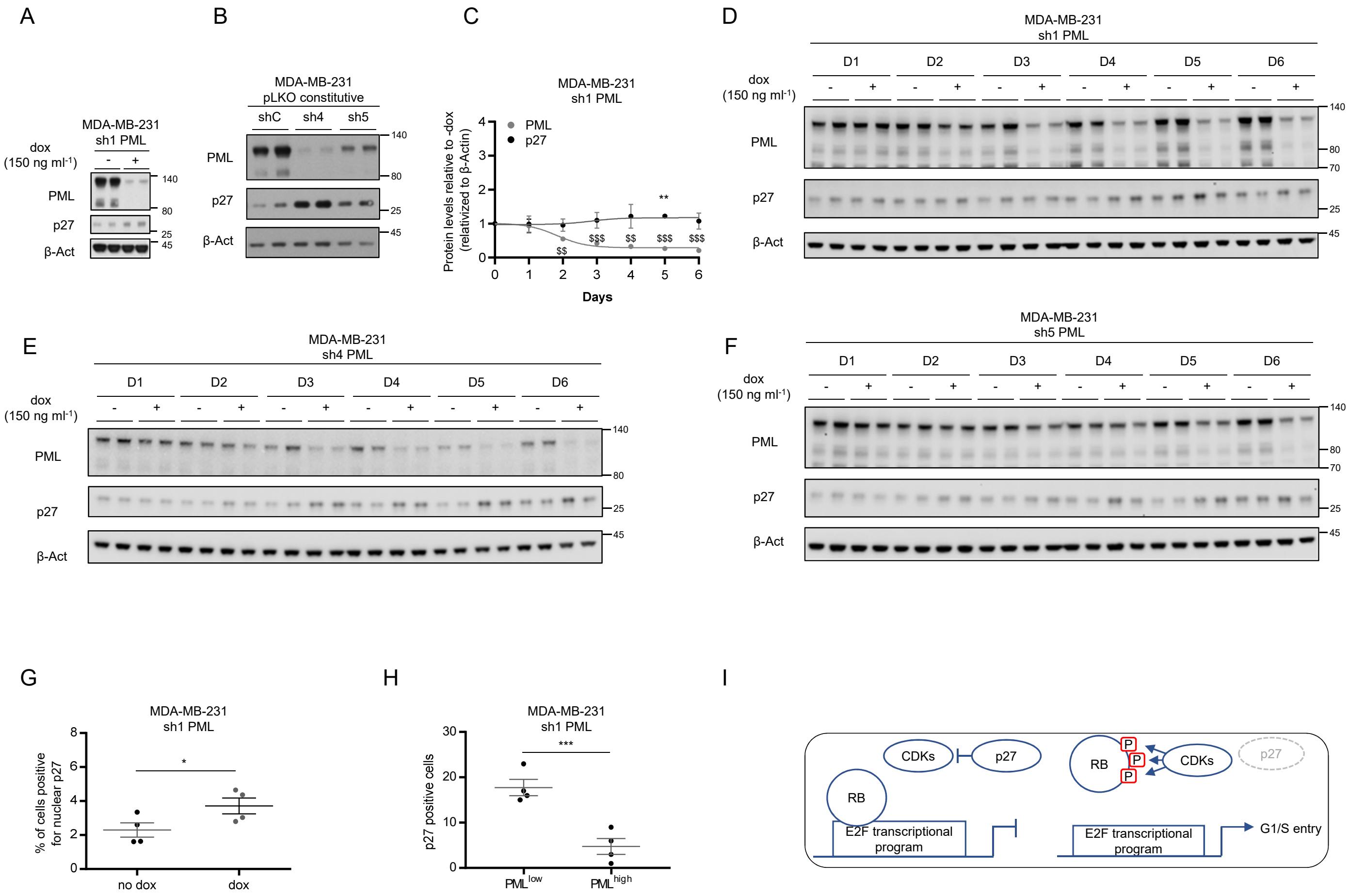
A
MDA-MB-231
sh1 PML
dox
(150 ng ml-1)
PML
p27
β-Act
B
MDA-MB-231
pLKO constitutive
shC
sh4
sh5
C
MDA-MB-231
sh1 PML
PML
p27
Protein levels relative to -dox (relativized to β-Actin)
Days
D
MDA-MB-231
sh1 PML
D1
D2
D3
D4
D5
D6
E
MDA-MB-231
sh4 PML
F
MDA-MB-231
sh5 PML
G
MDA-MB-231
sh1 PML
% of cells positive for nuclear p27
no dox
dox
H
p27 positive cells
PML low
PML high
I
CDKs
p27
RB
P
E2F transcriptional program
G1/S entry

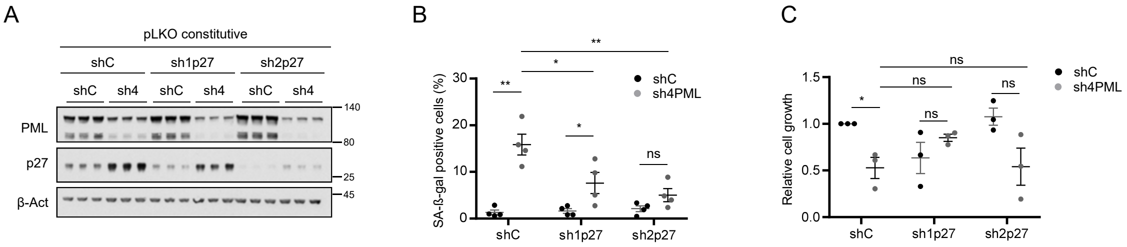
A
pLKO constitutive
shC
sh1p27
sh2p27
shC
sh4
shC
sh4
shC
sh4
PML
p27
β-Act
140
80
25
45
B
SA-ß-gal positive cells (%)
shC
sh4PML
shC
sh1p27
sh2p27
**
*
**
*
ns
C
Relative cell growth
shC
sh4PML
shC
sh1p27
sh2p27
*
ns
ns
ns
ns

Suppl. Figure 4

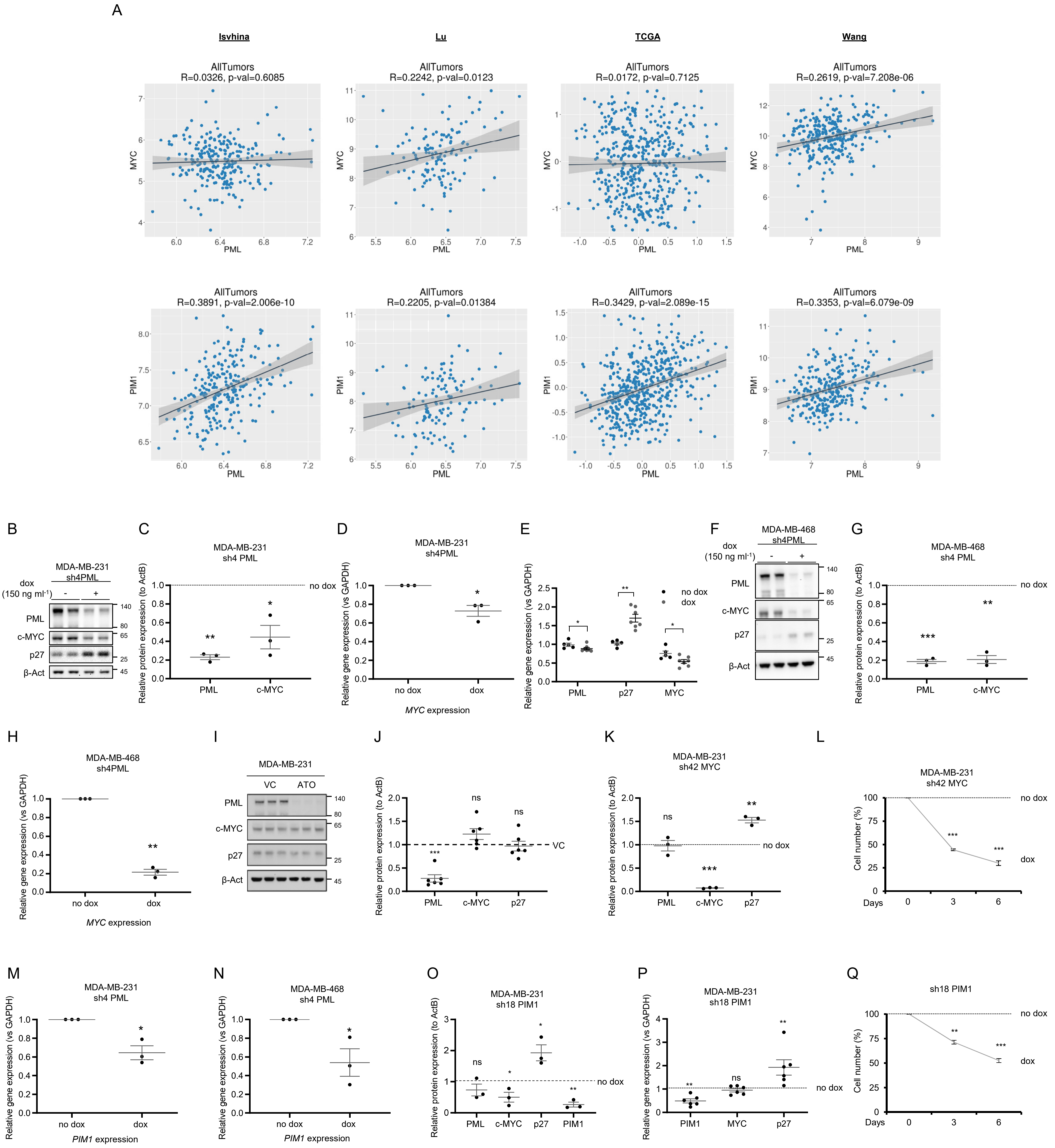

Supp. Figure 5

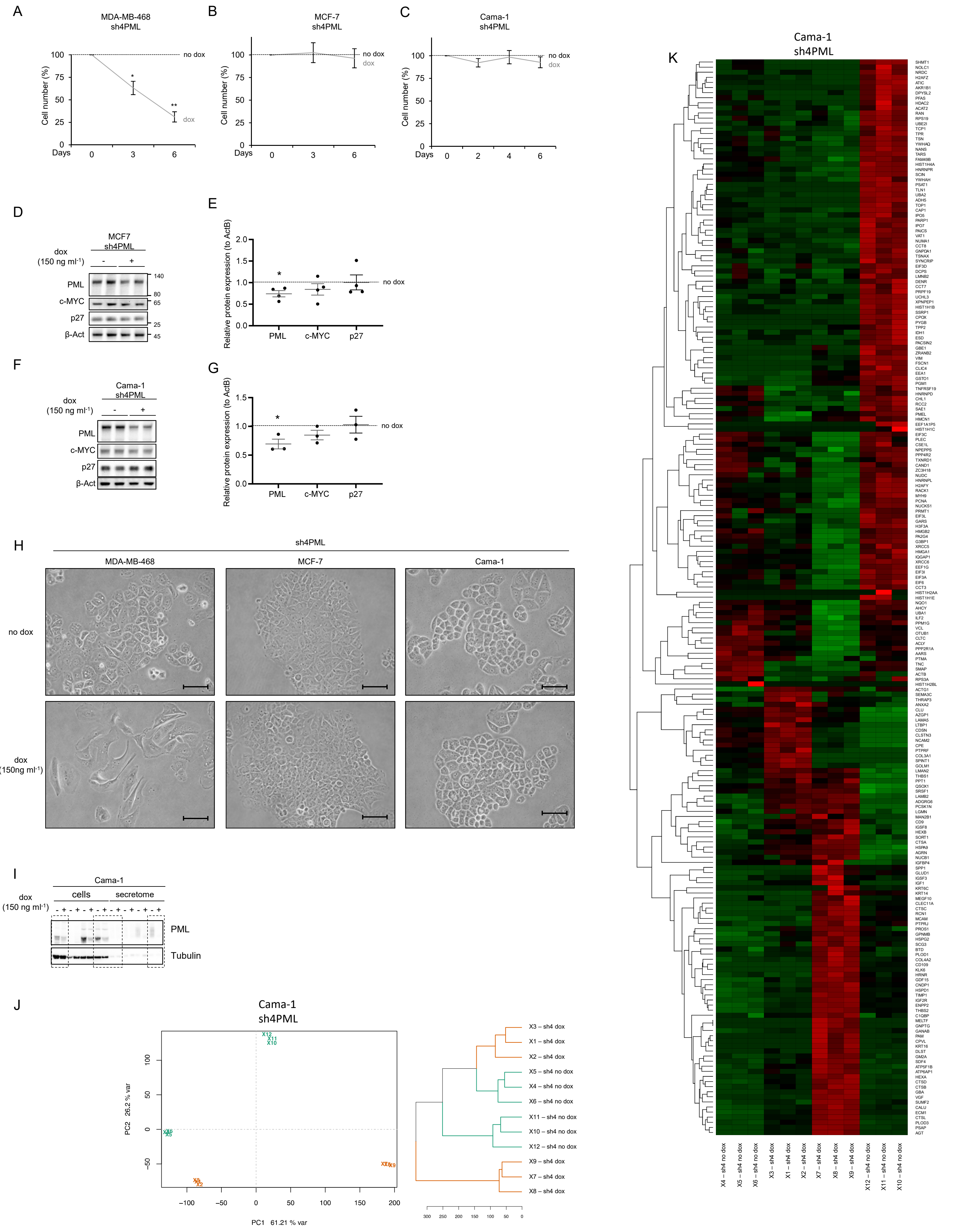

Supp. Figure 6

Figure 1A

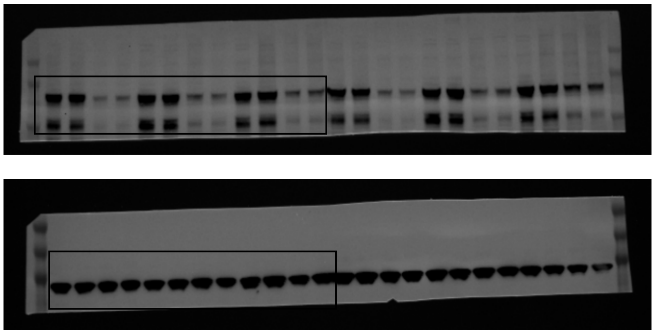

Figure 1G

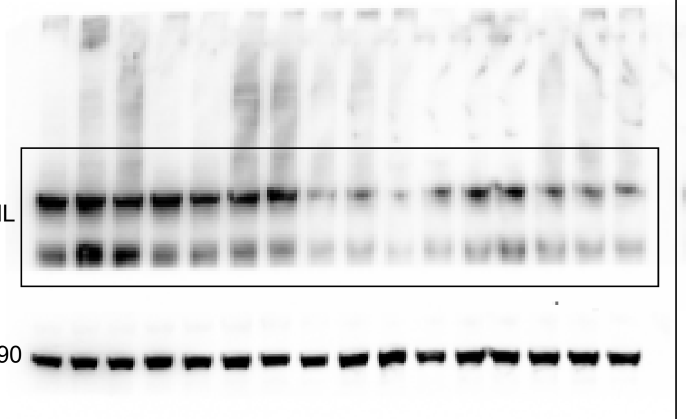

Figure S1A

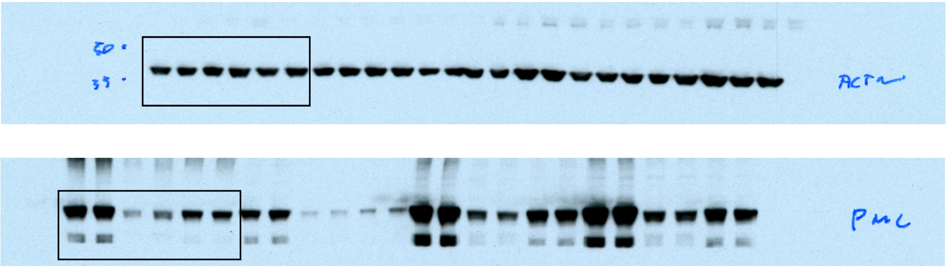

Figure S1H

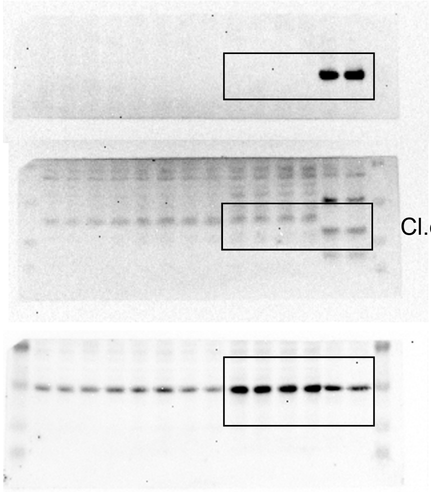

Figure S1I

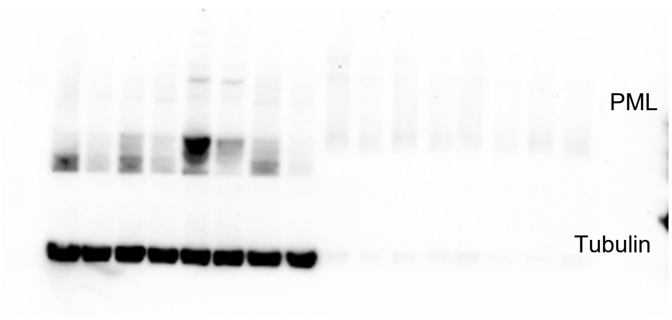

Figure S1m

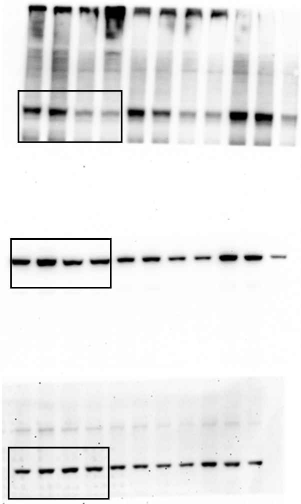

Figure 2A

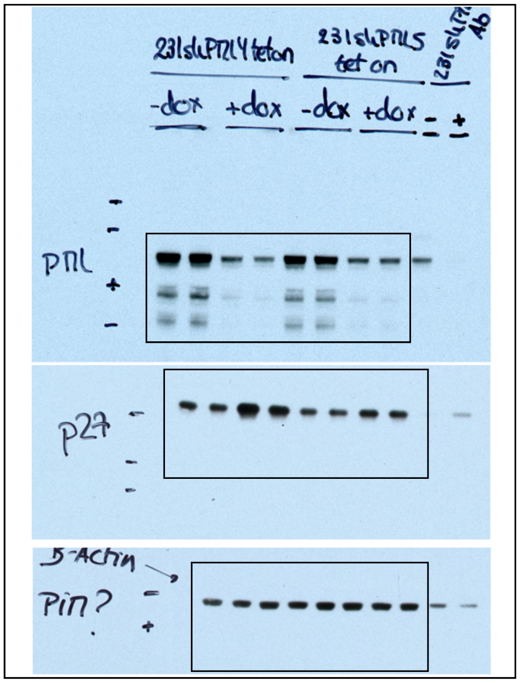

Figure 2H

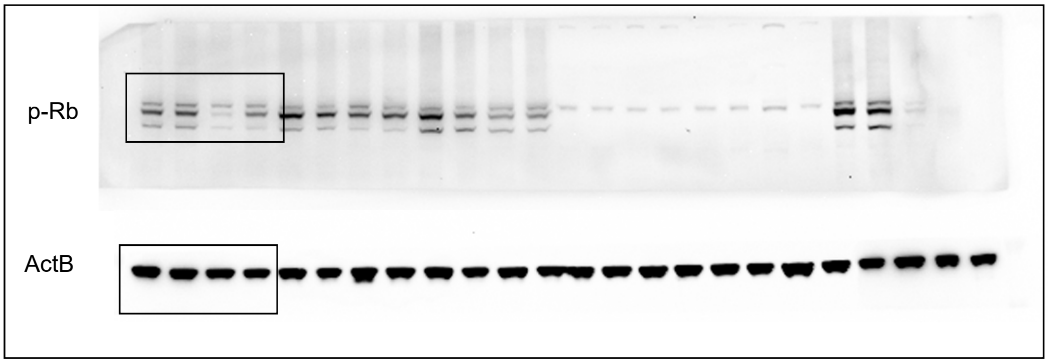

Figure 2J

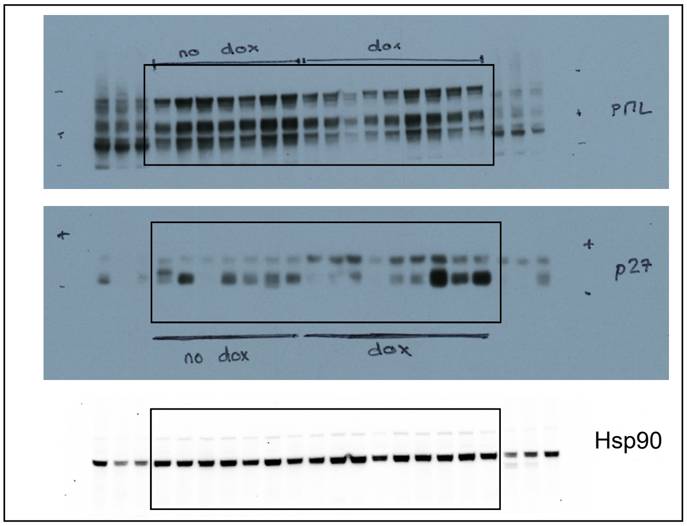

Figure S2A

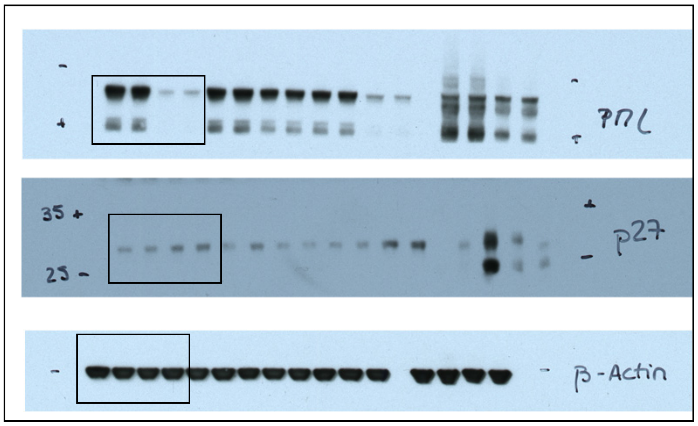

Figure S2B

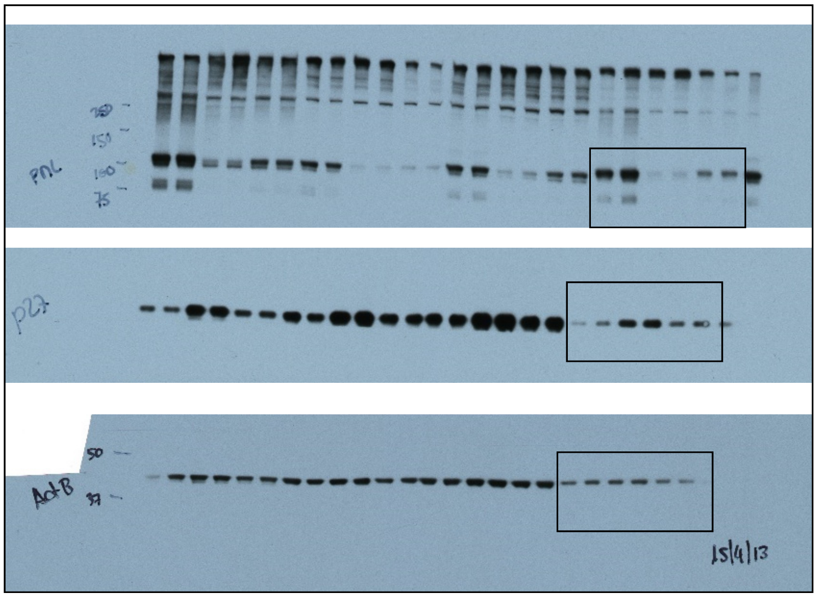

Figure S2D

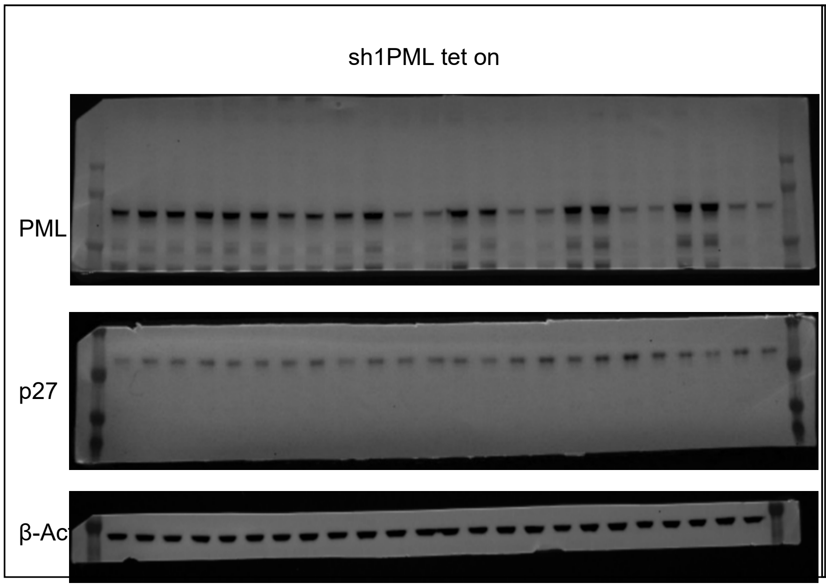

Figure S2E

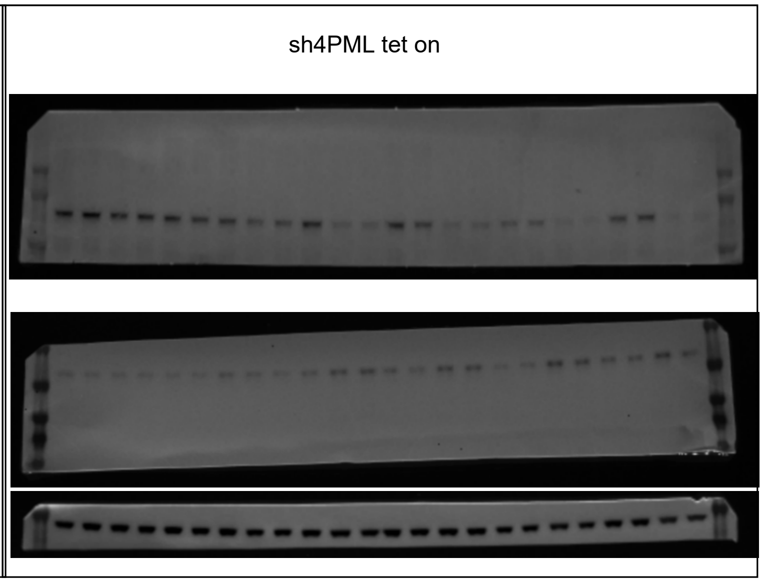

Figure S2F

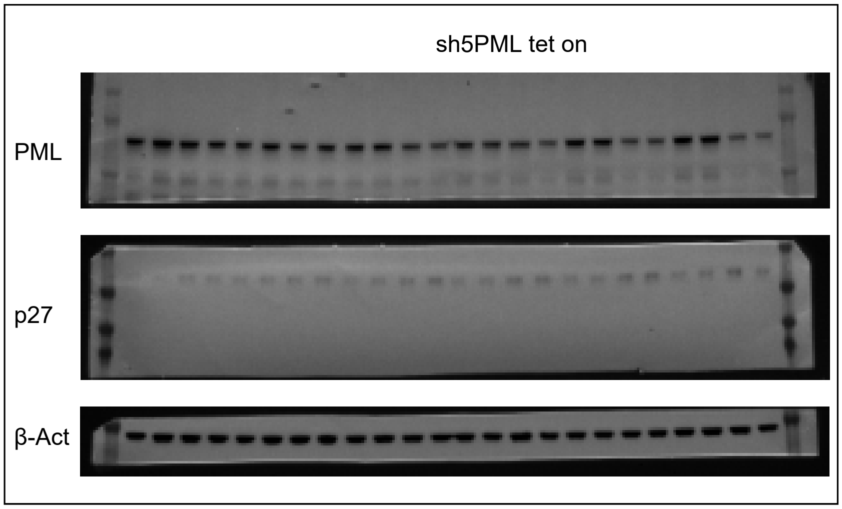

Figure 3B

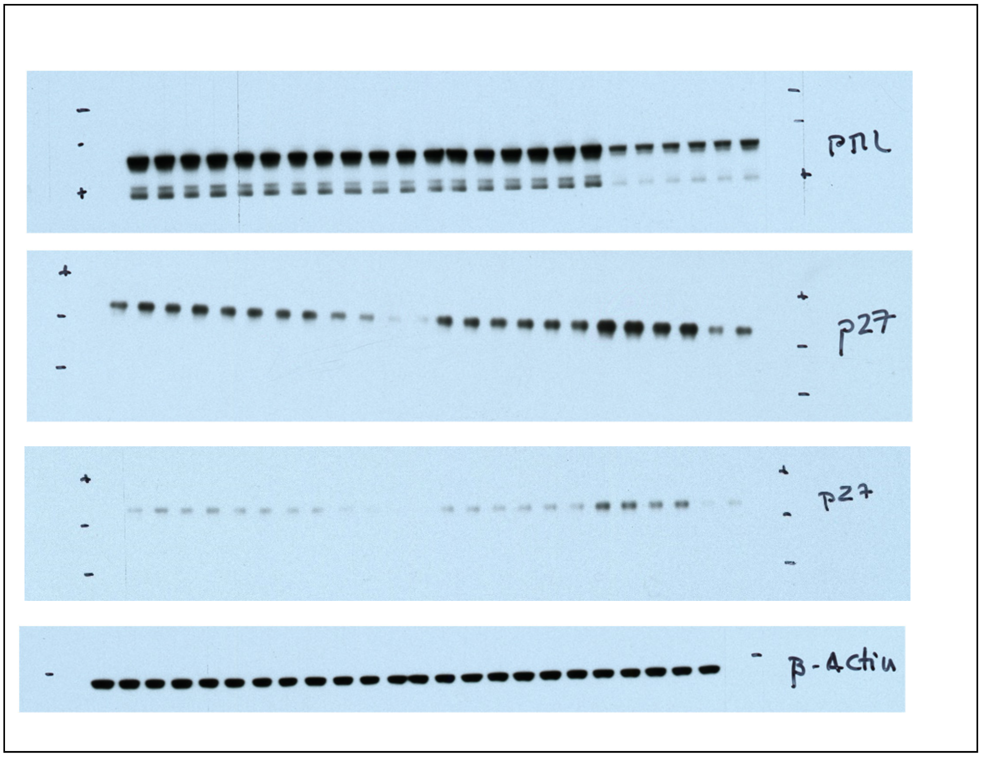

Figure S3A

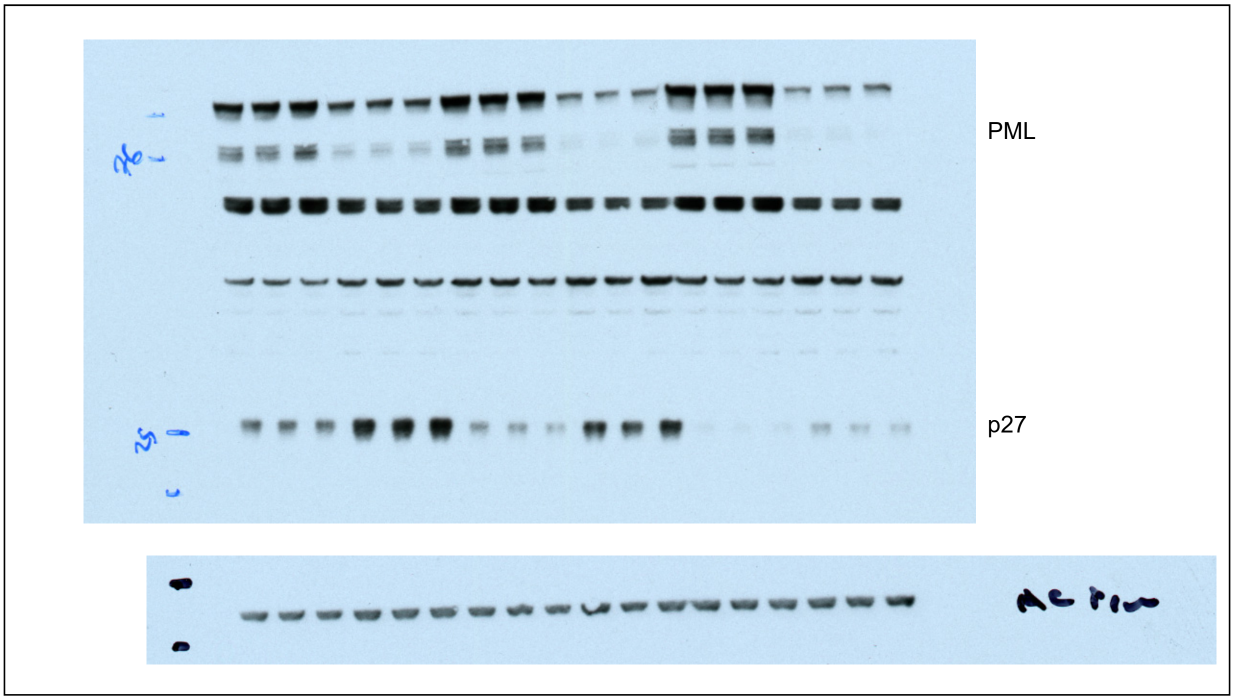

Supp. Figure 6 (cont)

Figure 4B

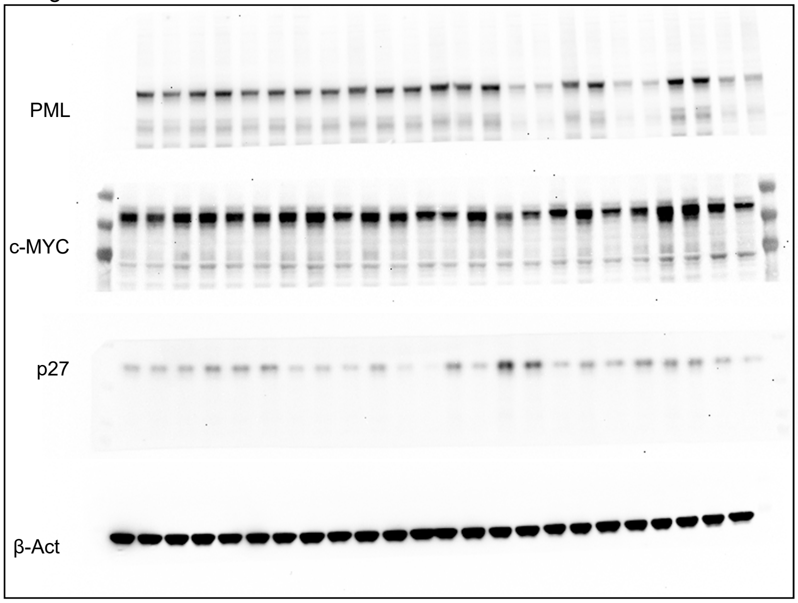

Figure 4E

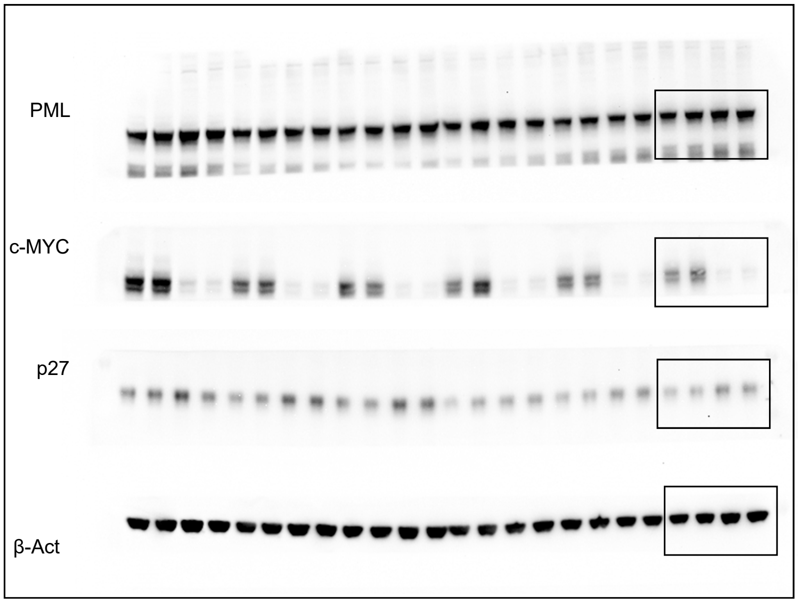

Figure 4I

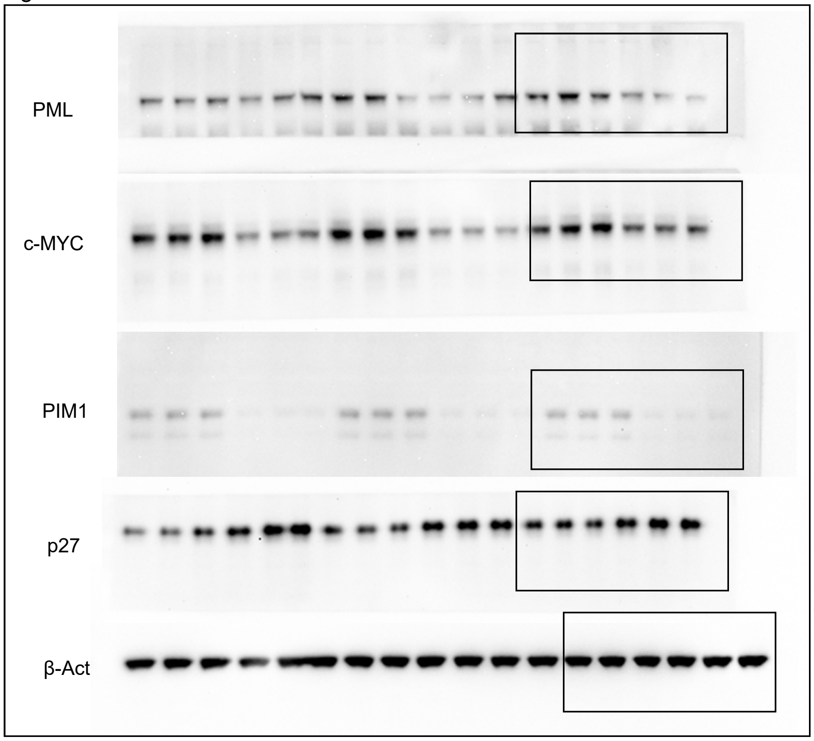

Figure S4B

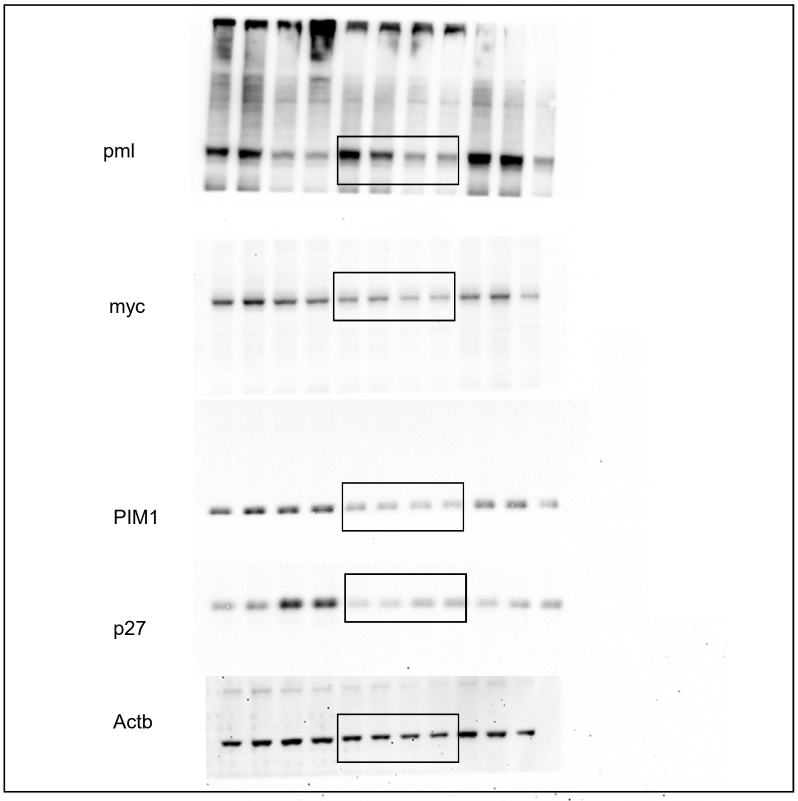

Figure S4F

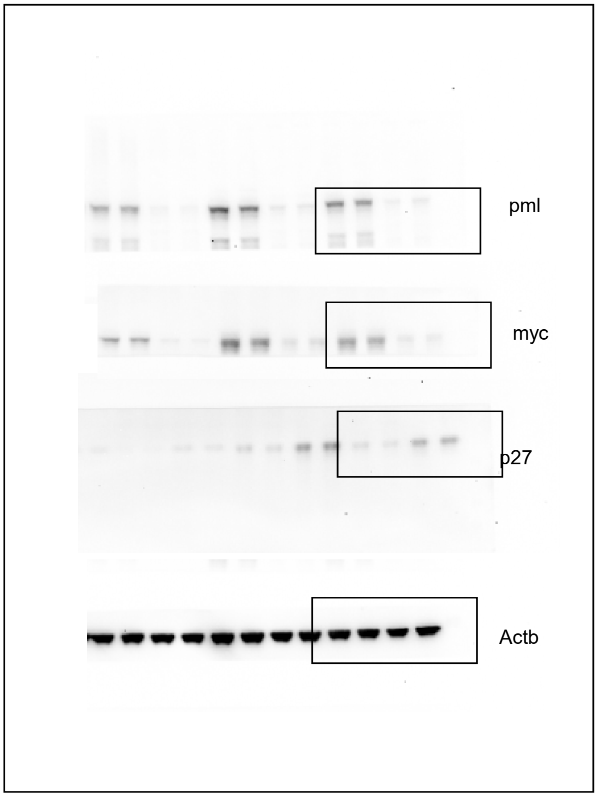

Figure S4I

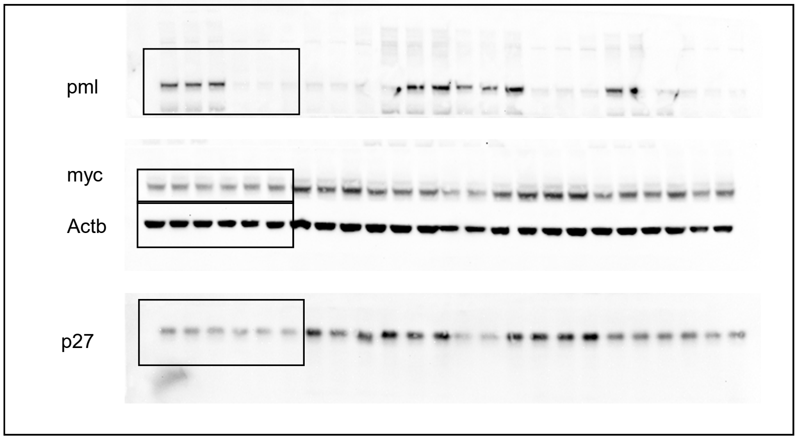

Figure 5A

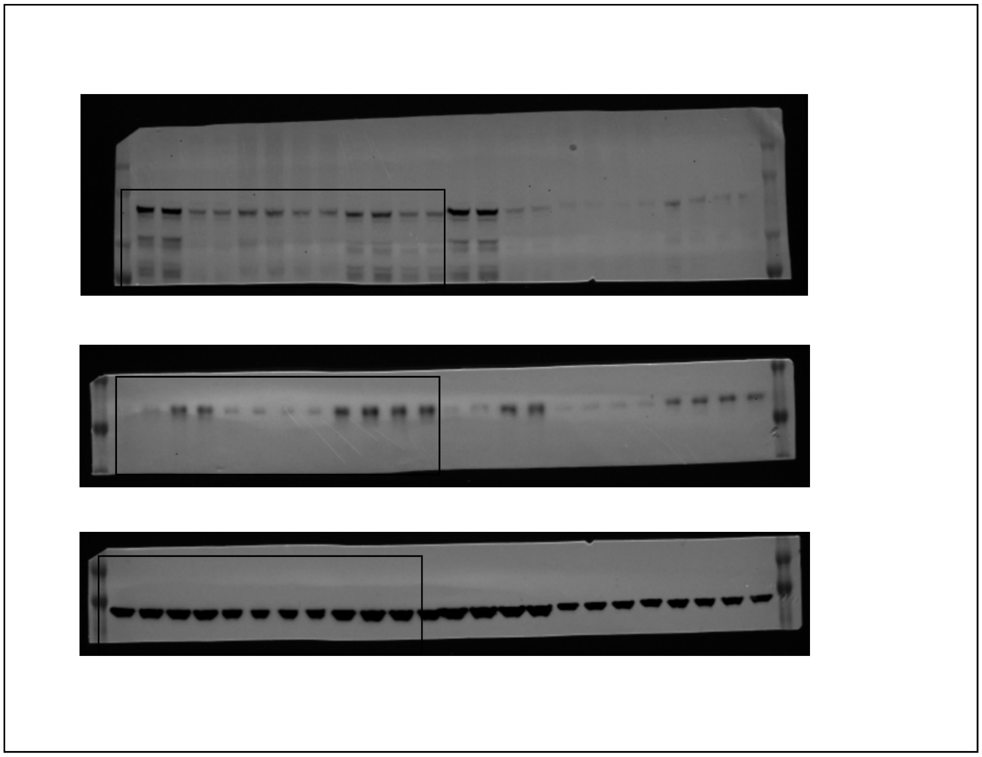

Figure S5D and S5F

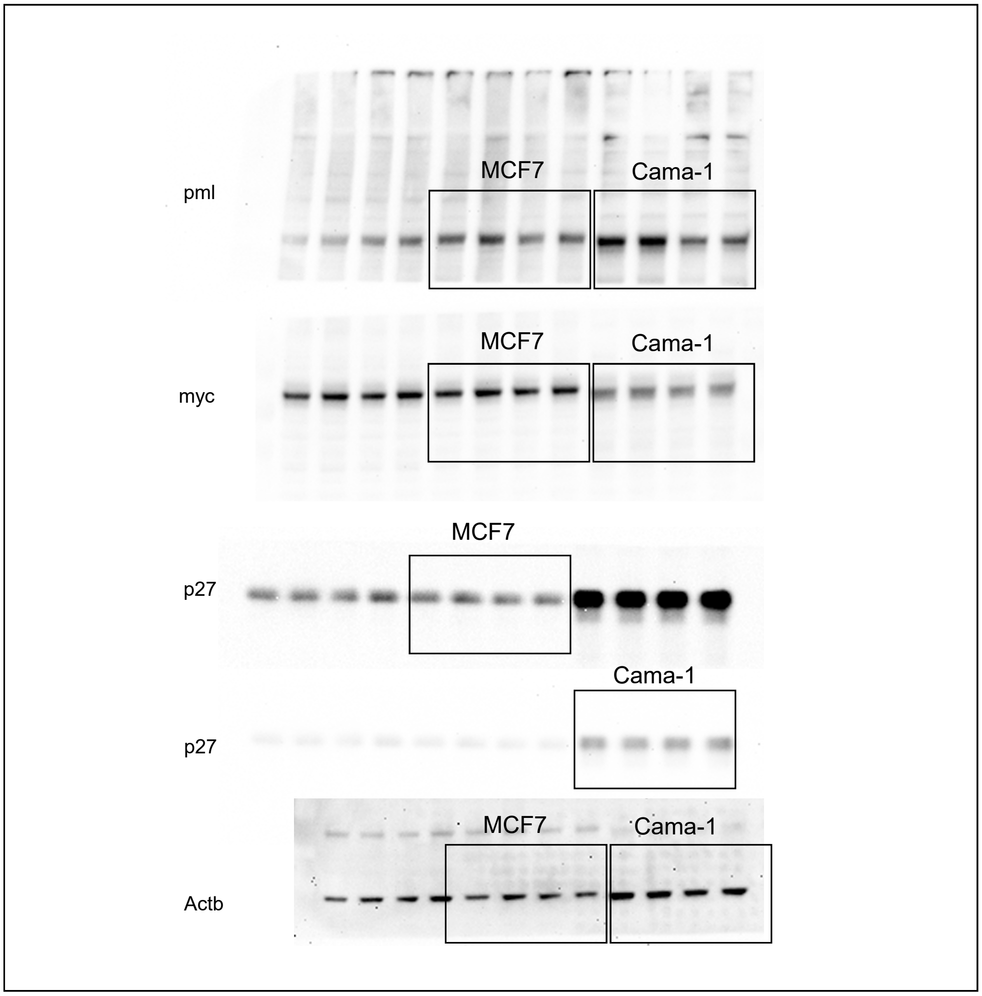

Figure S5I

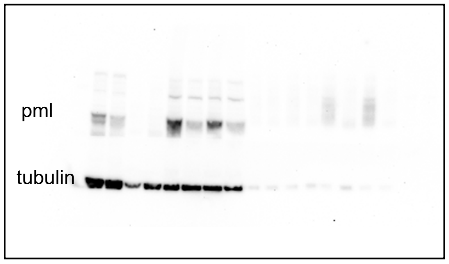

Supplement: Supplementary file 1 — Suppl Figures and legends [file 41418_2019_407_MOESM1_ESM.pdf]
